# Antioxidant genes of plants and fungal pathogens are distinctly regulated during disease development in different *Rhizoctonia solani* pathosystems

**DOI:** 10.1371/journal.pone.0192682

**Published:** 2018-02-21

**Authors:** Jamil Samsatly, Tanya R. Copley, Suha H. Jabaji

**Affiliations:** Plant Science Department, Macdonald Campus, McGill University, Ste-Anne-de-Bellevue, Canada; Leibniz-Institute of Vegetable and Ornamental Crops, GERMANY

## Abstract

Biotic stress, as a result of plant-pathogen interactions, induces the accumulation of reactive oxygen species in the cells, causing severe oxidative damage to plants and pathogens. To overcome this damage, both the host and pathogen have developed antioxidant systems to quench excess ROS and keep ROS production and scavenging systems under control. Data on ROS-scavenging systems in the necrotrophic plant pathogen *Rhizoctonia solani* are just emerging. We formerly identified vitamin B6 biosynthetic machinery of *R*. *solani* AG3 as a powerful antioxidant exhibiting a high ability to quench ROS, similar to *CATALASE* (*CAT*) and *GLUTATHIONE S-TRANSFERASE* (*GST*). Here, we provide evidence on the involvement of *R*. *solani* vitamin B6 biosynthetic pathway genes; *RsolPDX1* (**KF620111.1**), *RsolPDX2* (**KF620112.1**), and *RsolPLR* (**KJ395592.1**) in vitamin B6 *de novo* biosynthesis by yeast complementation assays. Since gene expression studies focusing on oxidative stress responses of both the plant and the pathogen following *R*. *solani* infection are very limited, this study is the first coexpression analysis of genes encoding vitamin B6, *CAT* and *GST* in plant and fungal tissues of three pathosystems during interaction of different AG groups of *R*. *solani* with their respective hosts. The findings indicate that distinct expression patterns of fungal and host antioxidant genes were correlated in necrotic tissues and their surrounding areas in each of the three *R*. *solani* pathosystems: potato sprout-*R*. *solani* AG3; soybean hypocotyl-*R*. *solani* AG4 and soybean leaves-*R*. *solani* AG1-IA interactions. Levels of ROS increased in all types of potato and soybean tissues, and in fungal hyphae following infection of *R*. *solani* AGs as determined by non-fluorescence and fluorescence methods using H_2_DCF-DA and DAB, respectively. Overall, we demonstrate that the co-expression and accumulation of certain plant and pathogen ROS-antioxidant related genes in each pathosystem are highlighted and might be critical during disease development from the plant’s point of view, and in pathogenicity and developing of infection structures from the fungal point of view.

## Introduction

The nectrotrophic fungus *Rhizoctonia solani* Kühn (teleomorph *Thanatephorus cucumeris*, Frank, Donk) is an economically devastating plant pathogen with a wide host range. *R*. *solani* is classified into fourteen anastomosis groups (AGs) based on hyphal fusion [1], and strains belonging to AG3 and AG4 are root infecting pathogens that cause damping-off and stem rot of potato (*Solanum tuberosum* L.) and soybean (*Glycine max* (L.) seedlings, respectively [2–5]. Isolates of the *R*. *solani* AG1-IA complex can infect aerial portions of the plant as in the case of soybean leaves causing rhizocotnia foliar blight (RFB) [3, 6, 7]. The common elements in disease development of *R*. *solani* isolates are the close association of fungal hyphae with the host epidermis forming branches known as infection cushions or aggregates, penetration of the epidermis, inter- and intracellular colonization and breakage of plant tissue by the production of hydrolytic enzymes, which eventually leads to the development of browning and necrosis associated with oxidative burst and death of tissue [4, 8]. Current cultural and chemical controls are not completely effective to manage Rhizoctonia diseases and the diseases remain a persistent problem. Furthermore, resistance to *R*. *solani* in any plant species does not exist.

One of the earliest plant responses following pathogen recognition is the hypersensitive response, leading to the production of reactive oxygen species, primarily superoxide (O_2_^-^) and H_2_O_2_, at the site of attempted invasion [9]. The produced ROS activates plant defense responses, including programmed cell death, or functions as second messengers in the induction of various plant defense-related genes [10, 11]. In the case of necrotrophic fungi, ROS plays a central role during their interaction with their plant hosts by stimulating the plant’s basal defense responses [12–15]. Several studies showed that the onset of basal resistance in plants to *R*. *solani* is tied with ROS-scavenging mechanisms, accumulation of metabolites related to vitamin B6 biosynthetic pathway, oxylipins production and cell wall bound phenolic compounds [16–21].

Different strategies of oxidative stress response systems are deployed by biotrophic and necrotrophic fungi during the infection process. Biotrophic pathogens such as rust fungi respond to oxidative stress by containing and suppressing the oxidative burst, while necrotrophic pathogens such as *Botrytis cincerea* rely on the exploitation of the oxidative burst in plants to its own advantage and in some cases contribute to it [22]. Hence, the ability of necrotrophic fungi to surpass or manipulate ROS-related plant defenses is crucial for disease progression, and ROS detoxification is essential to the sensitivity of the necrotrophic fungus while encountering its host. Detoxification systems, such as the NOX complex is a good example of how necrotrophic fungi such as *B*. *cinerea* and *Alternaria alternata* can sustain a reduced redox states within subcellular microenvironments [22]. In recent years, vitamin B6 was recognized as a strong antioxidant displaying a great capacity to quench ROS matching that of tocopherols or ascorbic acid, and may have a function in stress alleviation in fungi and plants [23, 24]. Data on ROS-scavenging systems in *R*. *solani* are just emerging. To date, the upregulation of *R*. *solani* genes particularly, *PYRIDOXAL REDUCTASE* (*PLR AKR8*; DW520695), and *PYRIDOXAL-5-PHOSPHATASES* and *TRANSAMINASES* of the vitamin B6 salvage biosynthetic pathway, was reported in *R*. *solani* hyphae in association with a mycoparasite or an antagonistic bacteria, respectively [25, 26].

In our previous publication, we fully characterized two genes of the *de novo* vitamin B6 biosynthetic pathway; *RsolPDX1* (KF620111.1) and *RsolPDX2* (KF620112.1) genes, and one gene *RsolPLR* (KJ395592.1) of the vitamin B6 salvage biosynthetic pathway of *R*. *solani* AG3. Upon exposure to the ROS-generating chemicals, paraquat and H_2_O_2,_ the vitamin B6 genes exhibited differential regulation and their transcript abundance levels were mostly higher than levels to the well-recognized antioxidant genes, *CATALASE* (*CAT*) and *GLUTATHIONE S-TRANSFERASE* (*GST*) [27]. These results implicated the role of vitamin B6 genes of the *de novo* and the salvage biosynthetic pathways function as antioxidants against oxidative stress [27].

Gene expression studies focusing on oxidative stress responses from both the plant and the pathogen sides during plant-*R*. *solani* interactions are very limited. Foley, Kidd [28] identified a number of wheat-derived and *R*. *solani* AG8-derived genes involved in ROS production and redox regulation, and whose expression was affected during infection. Their study highlighted the need to understand the ROS-scavenging system interplay of fungal and host derived genes involved in ROS/redox regulation at the site of infection and the surrounding areas. However our knowledge on the role of vitamin B6 genes as antioxidants in other AGs of *R*. *solani* is limited.

In this study, we first provided indirect evidence on the functionality of RsolPDX1 and RsolPDX2 of *R*. *solani* AG3, and their involvement in vitamin B6 *de novo* biosynthesis pathway via heterologous complementation of the yeast, *Saccharomyces cerevisiae* strains Δsnz1 and sno1Δ. Second, we showed that the antioxidant genes encoding vitamin B6 (i.e., *PDX*, *PLR*), *CAT* and, *GST* of *R*. *solani* AG3 and potato are differentially induced and transcriptionally regulated at the site of infection (i.e., necrotic tissues, and in the surrounding areas) during *R*. *solani* AG3-potato sprout interaction (pathosystem I). Third, we extended our study to investigate whether differential and spatial expression patterns and transcriptional regulation of vitamin B6 genes and other antioxidant genes also occur in the pathogen and host upon *R*. *solani* AG4 infection of soybean hypocotyl (pathosystem II) and also upon *R*. *solani* AG1-IA infection of soybean leaves (pathosystem III).

## Materials and methods

### Yeast complementation assays- heterologous expression of *R*. *solani* AG3 *PDX1* and *PDX2* and growth assays

Full-length clones of *RsolPDX1-*AG3 *and RsolPDX2-*AG3 were obtained by primer pairs (*RsolAG3-PDX1-CDS-*F/R and *RsolAG3-PDX2-CDS—*F/R) (Table 1) from cDNA of *R*. *solani* AG3 isolate Rs114 (ATCC 10183. The PCR products of the two genes were purified using the QIA quick PCR Purification Kit (Qiagen, Toronto, ON, Canada), and sub-cloned into the pDrive cloning vector (Qiagen) to obtain pDrive-*PDX1*-AG3 and pDrive-*PDX2*-AG3. The clones were digested with NotI and ligated into a NotI-digested yeast shuttle vector pFL61 (Addgene, Cambridge, MA, USA) [29] to obtain pFL61-*PDX1*-AG3 and pFL61-*PDX2*-AG3. All clones were sequenced to confirm the presence of the target genes. To determine whether *PDX1*-AG3 and *PDX2*-AG3 encode functional proteins, the *Saccharomyces cerevisiae* mutant strains (Δ*snz1*:disrupted *pdx1* and Δ*sno1*:disrupted *pdx2*), obtained from the European *Saccharomyces cerevisiae* Archive for functional analysis (EUROSCARF; http://wwwrz.uni-frankfurt.de/FB/fb16/mikro/EUROSCARF/), were transformed using a lithium acetate-based method [30] with the construct pFL61-expression vector or with pFL61 alone (negative control), and tested for their capacity to complement the defect function of the snz1 and sno1.

**Table 1:** List of primers used in this study.

Target organism	Primer	Sequence (5′ → 3′)	Annealing temperature (°C)	Amplicon size (bp)	Reference
	*PDX1* (Pyridoxine biosynthesis gene)				
**Soybean**	*GM*^*a*^*- PDX1*.*1-F*	TACAGTGTACGGCAACGGTGCA	59	235	XM_003542937.3
	*GM-PDX1*.*1-R*	GTCCTTGATGAGCTGCGGG			
	*GM-PDX1*.*2-F*	TACAGTCTACGGCAACGGCGCC	54	235	NM_001251112.2
	*GM-PDX1*.*2-R*	ATCCTTGATGAGCTGCGGA			
**Potato**	*ST*^*b*^-*PDX1*.*1-qF*	CTGTGACTATTCCTGTAAT	55	83	[37]
	*ST*- *PDX1*.*1-qR*	GTAATCTACTCCGATAGC			
	*ST*-*PDX1*.*2-qF*	TGCTCTAATCCTTACAAG	55	162	
	*ST-PDX1*.*2-qR*	GTAGGTCTCATCACTAAC			
***R*. *solani* AG3**	*5'GSP1-(PDX1)*	TGACGAACAGCCTCGACAACATTTCC	55	219	[27]
	*3'GSP2-(PDX1)*	TCCGTTTGTCTGTGGGGCTACATCTCTC			
***R*. *solani* AG4**	*Rsol*^*c*^*AG8-PDX1- F*	ATGATCAAAGAGATCGTGGAC	53	243	AVOZ01001318.1
	*RsolAG8-PDX1-R*	GATCATTGCTGCACCTTCGGA			
***R*. *solani* AG1-IA**	*RsolAG1-IA-PDX1-F*	ATCTCTGCCTACTACCAATG	51	131	AFRT01001225.1
	*RsolAG1-IA-PDX1-R*	TGCCCCCATCCGTGTTCTGAG			
	*PDX*2 (Pyridoxine biosynthesis gene)				
**Soybean**	*GM-PDX2-F*	TTAGGAGTGAAAGGTGTGGAG	53	328	XM_014774652.1
	*GM-PDX2-R*	TTTGGAGACGAGCTCTGGCAC			
**Potato**	*ST-PDX2-qF*	ATTCCAATCCTGCTATTC	55	185	[37]
	*ST-PDX2-qR*	CACAATATCAGAAGTTCCT			
***R*. *solani AG3***	*3'GSP1-(PDX2)*	AATCTTGCTTGCCTCTGGTGGTGTTG	55	225	[27]
	*5'GSP1-(PDX2)*	ATCCCATTAAATGGTCGGTCCTCATCA			
***R*. *solani AG4& R. solani AG1-IA***	*RsolAG8-PDX2-F*	ATTATATCACGCGTGACACC	53	207	JATN01000314.1
	*RsolAG8-PDX2-R*	GGCGCCTTCAACACCACCAGA			
	*PLR* (*PYRIDOXAL REDUCTASE*)				
**Soybean**	*GM-PLR-F*	TCCTCCAAAGCCTGAACCCGA	53	115	XM_003527250.3
	*GM-PLR-R*	TAGAAGGAGTTCGTTGGTGT			
**Potato**	*ST-PLR-F*	GCCGCCCAAACCCGAACCCGA	58	118	XM_006358747.2
	*ST-PLR-R*	AAGGAGGATCTCGTTAGTGT			
***R*. *solani* AG3**	*PLR AKR8 F*	GAAAGCCTCCTCTTGGAATCT	58	300	[25]
	*PLR AKR8 R*	GGGTAAGATTGGATCGATTGGG			
***R*. *solani* AG4 & *R*. *solani* AG1-IA**	*RsolAG1-IA-PLR-F*	AAGGGGCACTAACCGGAAAGC	58	194	AFRT01000983.1
	*RsolAG1-IA-PLR-R*	ATGTTGCCCGAGAGAGGAGAC			
	*CATALASE*				
**Soybean**	*GM-CAT*^*d*^*-F*	ACATGTTCACCTTTTTATTTG	47	149	NM_001253092
	*GM-CAT-R*	CTTTATACCACTAGTGGTCTT			
**Potato**	*ST-CAT-F*	ATGTTCACTTTCCTCTTCGAC	52	147	AY442179.2
	*ST-CAT-R*	CTTGACACCACATGTGGGCTT			
***R*. *solani* AG3**	*RsolAG3-CAT-F*	ACCAGAAGTGTTAGTCCAGCGG	56	190	[27]
	*RsolAG3-CAT-R*	CATCCGGTCACAGCAGCGTA			
***R*. *solani*** AG4& ***R*. *solani* AG1-IA**	*RsolAG1-IA-CAT-F*	TGCGGGACTCATGCTGCTCTC	55–54	114	AFRT01002805.1
	*RsolAG1-IA-CAT-R*	TCCTTGGTGCCTTTCCTATCC			
	*GLUTATHIONE S-TRANSFERASES*				
**Soybean**	*GM-GST* ^*e*^*-F*	TCCATTTGGGATGAGGGTCAG	53	208	NM_001251762
	*GM-GST-R*	ATTTCTGTCATTCCAAACCTCC			
**Potato**	*ST-GST-F*	TCCTTTTAGCCATAGAGTTGA	51	200	POTPR1A
	*ST-GST-R*	CTTCAAATGCCTCATCAATGC			
***R*. *solani* AG3**	*RsolAG3-GST-F*	AGAAGACGAGGCAAATGCGA	57	256	[27]
	*RsolAG3-GST-R*	ATCTCTTCAACCGCCTTCCAGT			
***R*. *solani* AG4 *& R*. *solani* AG1-IA**	*RsolAG1-IA-GST-F*	TACAAACGCATACCTTATCGC	53	217	ELU40094.1
	*RsolAG1-IA-GST -R*	TTGGAGCCGGGTAAGTTTTG			
	*Reference genes*				
**Soybean**	*UKN2*^*f*^ *_F*	GCCTCTGGATACCTGCTCAAG	58	79	[38]
	*UKN2_R*	ACCTCCTCCTCAAACTCCTCTG			
**Potato**	*ST-Actin7-qF*	GGCTATGTATGTTGCTAT	55	186	[37]
	*ST-Actin7-qR*	ATCTTCATCAGGTTATCAG			
***R*. *solani* AG3**	*G3PDH*^*g*^ *-F*	GGTATTATTGGATACACTGA	55	129	[25]
	*G3PDH -R*	TTAAGCCTCAGCGTCTTTCT			
***R*. *solani* AG1-IA**	*RsolH3*^*h*^ *_F*	CTTCCAATCATCGGCAGTCCTC	52	76	[34]
	*RsolH3 _R*	ATTGGTATCTTCGAACAAAGACACGAG			
***R*. *solani* AG4**	*GM-RS-4*^*i*^	CGGTTCATCTGCATTTACCTT	55	88	[39]
	*GM-RS3-R*	AGTGTTATGCTTGGTTCCACT			
	*Transformation*				
***R*. *solani* AG3**	*RsolAG3-PDX1-CDS-F* *RsolAG3-PDX1-CDS-R*	GCGGCCGCATCATGTCTGCTCCTGTGTC GCGGCCGCCACTCACATAGTCAGCCCAA	55	969	KF620111.1
***R*. *solani* AG3**	*RsolAG3-PDX2-CDS-F*	GCGGCCGCTCGATGACTAGAACTGAAAC	55	747	KF620112.1
	*RsolAG3-PDX2-CDS-R*	GCGGCCGCGGTTCACTTGGCAAAGCTC			

a Glycine max

b Solanum tuberosum

c Rhizoctonia solani

d Catalase

e Glutathione S-Transferase

f Hypothetical protein unknown 2

g G3PDH: Glyceraldehyde-3-phosphatedehydrogenasegen

h Histone 3

i ITS1 of 5.8S rRNA

For vitamin B6 growth-dependence assays: Yeast cultures grown to exponential phase (2 × 10^7^ cells mL^−1^) followed by two serial 10-fold dilutions corresponding to the wild-type BY4742, and transformants were spotted on minimal selective SC medium (Difco Laboratories, MI, USA) medium lacking vitamin B6. The mutants, Δsnz1 and Δsno1 are sensitive to the superoxide generator menadione, and this sensitivity is corrected by the addition of vitamin B6 [31]. Transformed yeast cells were grown on minimal SC medium to the post-diauxic phase for 20h followed by a treatment with 40 mM menadione (-Aldrich Canada, Mississauga, ON, Canada) for 3 h in the presence or absence of 2μg mL^−1^ of vitamin B6 and spotted on SC medium lacking Uracil and vitamin B6. Pictures were captured from plates that were incubated for 3 d at 30°C [32].

### Biological material and inoculation

#### Fungal material

Starter cultures of *R*. *solani* AG3 isolate Rs114 (ATCC 10183), AG4 isolate A76 obtained from M. Cubeta, North Carolina University, USA, and AG1-IA strain ROS-2A4 obtained from P. Ceresini, University of São Paulo State (UNESP), Brazil were recovered from cryogenic storage at −80°C on potato dextrose agar (PDA; Difco Laboratories) and grown for 3 days at 24°C in the dark and used as a source of inoculum for plant infection. Cultures of *R*. *solani* strains grown on half-strength PDA overlaid with cellophane membrane were used for the visualization of ROS in their hyphae.

#### Plant material

Certified potato tubers (*Solanum tuberosum* var. Russet Burbank, susceptible to *R*. *solani* AG3), obtained from the Potato Research Center (NB, Canada) and propagated at the Macdonald Campus Farm (Ste. Anne de Bellevue, Canada), were kindly provided by D.J. Donnelly (McGill University). Potato tubers were grown without light at 4°C to induce sprout formation. For sprout induction, uniform-sized tubers were surface sterilised and sprouted for 12 days under controlled conditions following the methods of Aliferis and Jabaji [17], Chamoun, Samsatly [33]. Prevention of browning of the sprout tips was achieved by applying 1.5 mL solution of CaNO_3_ (0.5M) to sprouts of 3 cm in length. Inoculation of sprouts was performed when sprouts were 8 cm in length.

Soybean (*Glycine max*) cv. Williams 82 seeds, a susceptible cultivar to *R*. *solani* AG-4, *were* sterilized according to Aliferis, Faubert [16]. Pre-germinated seeds were planted in Cone-tainers® (Stuewe & Sons, Inc., Oregon, USA) filled with 130 mL of sterile turf:perlite (1:1, v/v) and incubated in the dark in a growth chamber in order to produce etiolated seedlings with longer lengths of hypocotyls (approximately 15 cm in height). Hypocotyls were inoculated with AG4 isolate A76 as previously published [16].

For unifoliate leaf inoculation with *R*. *solani* AG1-IA, pre-germinated soybean seeds in Cone-tainers® as described above were grown under controlled conditions of temperature, humidity and light following the method of Copley, Aliferis [34]. Fully expanded soybean unifoliate leaves were detached from 2 week-old plants, and used for inoculation.

### Inoculation of potato sprouts, soybean hypocotyls and soybean leaves

Excised sprouts and etiolated hypocotyls were arranged horizontally in sterile Pyrex trays (40 cm × 26 cm) lined with wet sterile Whatman No. 1 paper. Each treatment replicate consisted of 4 excised potato sprouts or 4 etiolated hypocotyls. There were 3 biological replicates per treatment. Inoculation was performed by sandwiching horizontally the sprouts (8 cm in length) or hypocotyls (15 cm in length) of each treatment replicate between two PDA strips (2 cm × 8 cm) of a 3-day-old *R*. *solani* AG3 Rs114 or *R*. *solani* AG4 isolate A76 cultures, respectively, with the bottom edge of the strips placed at 4 cm from the basal part of the potato sprouts or hypocotyl sprouts according to the method of Aliferis and Jabaji [17], Chamoun, Samsatly [33]. Potato sprouts or soybean hypocotyls sandwiched with sterile PDA strips served as controls. Moist, sterile absorbent cotton wool was placed at the base of the sprouts or the hypocotyls of each replicate treatment to maintain humidity. Pyrex trays were sealed with Saran Wrap, and placed in a growth chamber under photosynthetically inactive black light (365 nm) at 24°C.

Fully expanded unifoliate soybean leaves were placed into sterile Pyrex trays as described above. Inoculation was performed by placing PDA plugs (5 mm) of 3-day-old *R*. *solani* AG1-IA culture disc, or with sterile PDA alone (control) at the center of leaves. Each replicate treatment consisted of 5 unifoliate leaves. Trays were wrapped with plastic membrane, placed in growth chambers under conditions of day/night temperatures, light cycles and humidity as we previously described in Copley, Aliferis [34].

**Fig 1:**
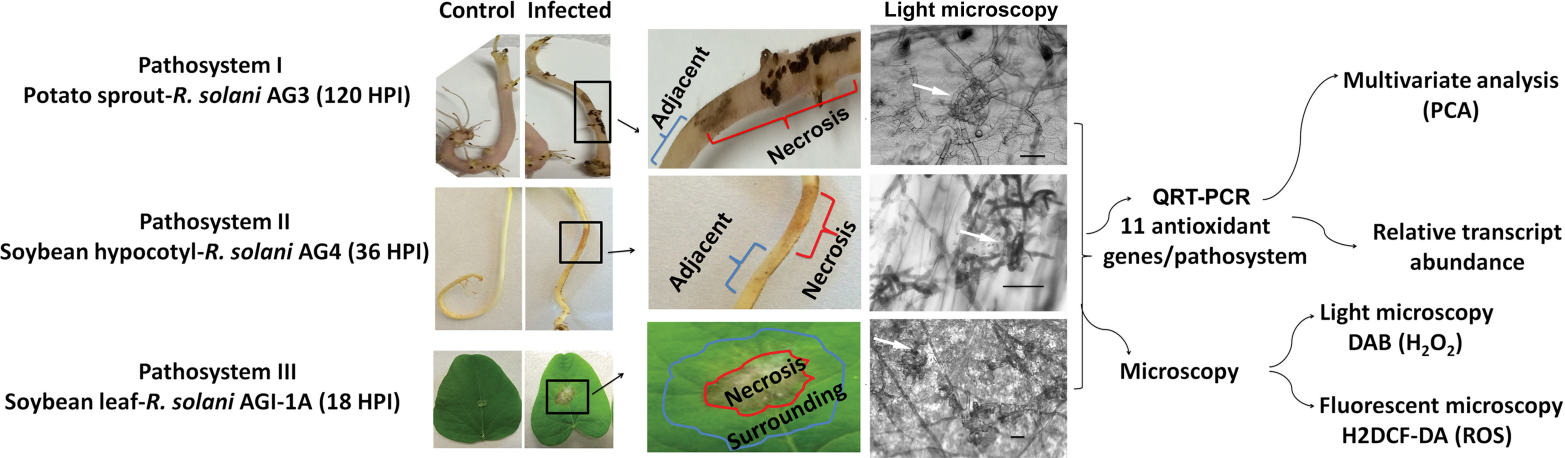
Flow chart of the experimental set up for disease development in the three pathosystems. I. potato sprouts-*R*. *solani* AG3; II. soybean hypocotyls*-R*. *solani* AG4; III. soybean leaves*-R*. *solani* AG1-IA. Sprouts and hypocotyls in pathosystems I and II, were sandwiched between two PDA strips of a 3-day-old *R*. *solani* AG3 or AG4 cultures, respectively. Sprouts or hypocotyls sandwiched with sterile PDA strips served as controls. Leaves in pathosystem III were inoculated with 3-day-old plugs of *R*. *solani* AG1-IA. Samples were taken at 120 HPI (Hour post-inoculation) (pathosystem I), 36 HPI (pathosystem II), and 18 HPI (pathosystem III) from necrotic lesions and adjacent or surrounding areas. Corresponding segments were taken from controls. Typical initials of infection cushions with mycelial mass aggregates are clearly visible under the light microscope (white arrows). Transcriptional abundance of 11 antioxidant genes from both the plant and pathogen of each pathosystem was analyzed in necrotic tissues and their surrounding areas followed by multivariate analysis (PCA). The three pathosystems were examined for ROS accumulation using fluorescent (H_2_DCF-DA) and DAB staining methods in control and infected sprouts, hypocotyls and leaves along with mycelia of control *R*. *solani*. Bar = 50 μm.

PDA strips or plugs were removed 120 h, 36h and 18h post-inoculation (HPI) of plant tissue to reveal visible necrotic lesions with infection zones on sprouts, hypocotyls and leaves, respectively. These time points were selected in order to capture the onset of infection cushions and the development of mycelial aggregates that were stereoscopically confirmed (**Fig 1**). Necrotic tissues containing infection zones plus a one-cm area away from necrotic tissues were harvested. Similar areas and amounts of tissues were harvested from control samples. Harvested tissues were flash-frozen with liquid nitrogen and stored at −80°C. Each treatment (infected or control) consisted of three biological replications.

### RNA extraction, primer design and quantitative RT-PCR

Total RNA was isolated from 100 mg of flash frozen pulverized tissue obtained from the necrotic lesions and their surrounding areas of respective treatments and corresponding areas of control soybean hypocotyls, leaves, or potato sprouts. Extraction was done using the TRIZOL reagent (Generay Biotech, Shanghai, China) following the manufacturer's instructions. RNA concentration and purity was spectrophotometry measured using ND1000 (NanoDrop, Wilmington, Delaware), and RNA quality was verified by gel electrophoresis. RNA (500 ng) was reverse transcribed using the Quantitect Reverse transcriptase kit™ (Qiagen). QRT-PCR assays were conducted on fungal and plant target antioxidant genes encoding *GST*, *CAT*, the *de novo* vitamin B6 biosynthesis genes; a synthase (*PDX1*) and a *glutaminase*, (*PDX2*), and the vitamin B6 salvage pathway encoding gene, *PYRIDOXAL REDUCTASE* (*PLR*) and appropriate internal reference genes for the three pathosystems (Table 1*)* using Stratagene Mx3000 (Stratagene, Cedar Creek, USA). Primer names were preceded by the plant or fungus abbreviation: *GM*, *Glycine* max, *ST*, *Solanum tuberosum*, or *Rsol*, *R*. *solani*. Primer sets were designed based on sequences from NCBI, and were checked for specificity to amplify only their target gene. QRT-PCR conditions were optimized for each primer set, and products were confirmed by sequencing. For primer pairs that were used on more than one AG, they were checked for their ability to amplify with comparable efficiencies in the different pathosystems. Reverse transcription PCR assays were performed on three biological replicates and two technical replicates. PCR assay conditions were performed as previously described [27, 35] using suitable annealing temperature for each primer pair (Table 1). In all QRT-PCR assays, routine negative and positive controls were performed at every run. No template control served as negative control. Also, cDNA of uninfected plant host or *R*. *solani* alone served as negative controls when amplifying fungal genes or plant genes, respectively. Positive controls for each run consisted of cDNA of *R*. *solani* alone or plant host alone when amplifying the fungal or plant genes, respectively. The relative transcript abundance levels of the plant and fungal-derived genes were estimated and normalized against their respective reference genes according to Zhao and Fernald [36]. In the case of *R*. *solani* AG-3, expression of the antioxidant genes was normalized using G3PDH. This choice was based on the lowest coefficient variation when compared to Tubulin and Histone using the statistical tool Bestkeeper (http://www.gene-quantifiaction.info).

### Statistical analyses

Significance of the relative transcript abundance between treatments and controls were analyzed by two-way analysis of variance (ANOVA), and when necessary by least significant differences (LSD) at *P* < 0.05 using the SPSS statistical package v. 22.0, (IBM Corp., Armonk, NY, USA). Transcript changes were deemed statistically and biologically significant if *P*<0.05 and fold changes were > +1.5 or > -1.5.

In order to find potential correlations and trends between relative abundance of plant and fungal genes expressed in necrotic tissues and their surrounding areas of each pathosystem, a multivariate analysis of the data was performed using the SIMCA-P+ v.12.0 software (Umetrics, MKS Instruments Inc., Andover, MA, USA). The data matrix consisted of biological replicates of control plant tissues, and tissues of necrotic lesions and areas surrounding them (columns; X variables) and the variables for relative transcript abundance of antioxidant genes (rows; Y variables). Data were obtained from the analysis of relative transcript abundance of 11 genes for potato sprouts*-R*. *solani* AG3, soybean leaves-*R*. *solani* AG1-IA and soybean hypocotyls-*R*. *solani* AG4 [11 rows x 9 columns]. For the evaluation of data and detection of outliers, principal component analysis (PCA) was performed. To determine which of the genes were most affected in each pathosystem, PCA loading coefficient plot for the effect of the relative transcript abundance of genes on tissue type, and a loading biplot was built for the visualization of correlations between the X and Y variables using the pc (corr) (i.e., correlation scaled loadings of the correlation between Y variables and X scores based on the variable importance of X as scores and loadings) setting in SIMCA-P+. For data normalization, mean-centering and Pareto [PAR] scaling were used.

### Optical and fluorescence microscopy

Association of the detected ROS that accumulated during disease development, with changes in the transcript abundance of the genes was visualized during disease development in the three pathosystems. Cellular and extracellular ROS accumulation was visualized by 2′,7′-dichlorodihydrofluorescein diacetate (H_2_DCF-DA) and 3,3′-diaminobenzidine (DAB) staining methods in control and infected soybean leaves and hypocotyls, and potato sprouts, along with mycelia of control *R*. *solani* AG1-IA, AG4, AG3 grown on half-strength PDA overlaid with cellophane membrane. For ROS detection, samples were incubated with 10 μM H_2_DCF-DA, a specific ROS molecular-detection probe, in H_2_O for 30 min. Samples were then washed with pre-warmed (28°C) H_2_O for 30 min to remove the non-internalized probe. To visualize H_2_O_2_ accumulation *in situ*, DAB staining was performed, by treating the samples DAB as described in Pogany, von Rad [40] with some modifications. Whole leaves of soybean were treated with DAB for 24h, whereas thin shavings (approximately 2 x 0.5 cm) of soybean hypocotyls and potato sprouts were obtained with a blade and treated for 4h. All samples were cleared with saturated 15.1 M of chloral hydrate solution, and examined under Zeiss SteREO Discovery.V20 microscope (Carl Zeiss Canada Ltd., Toronto, Ontario, Canada). Fluorescence detection from plant and fungal tissues were read at an excitation wavelength of 470 nm using a GFP filter.

## Results

### Functional characterization of *RsolPDX1* and *RsolPDX2* by yeast heterologous complementation provided proof of the ability of *R*. *solani* AG3 *PDX1* and *PDX2* to encode functional enzymes

**Fig 2:**
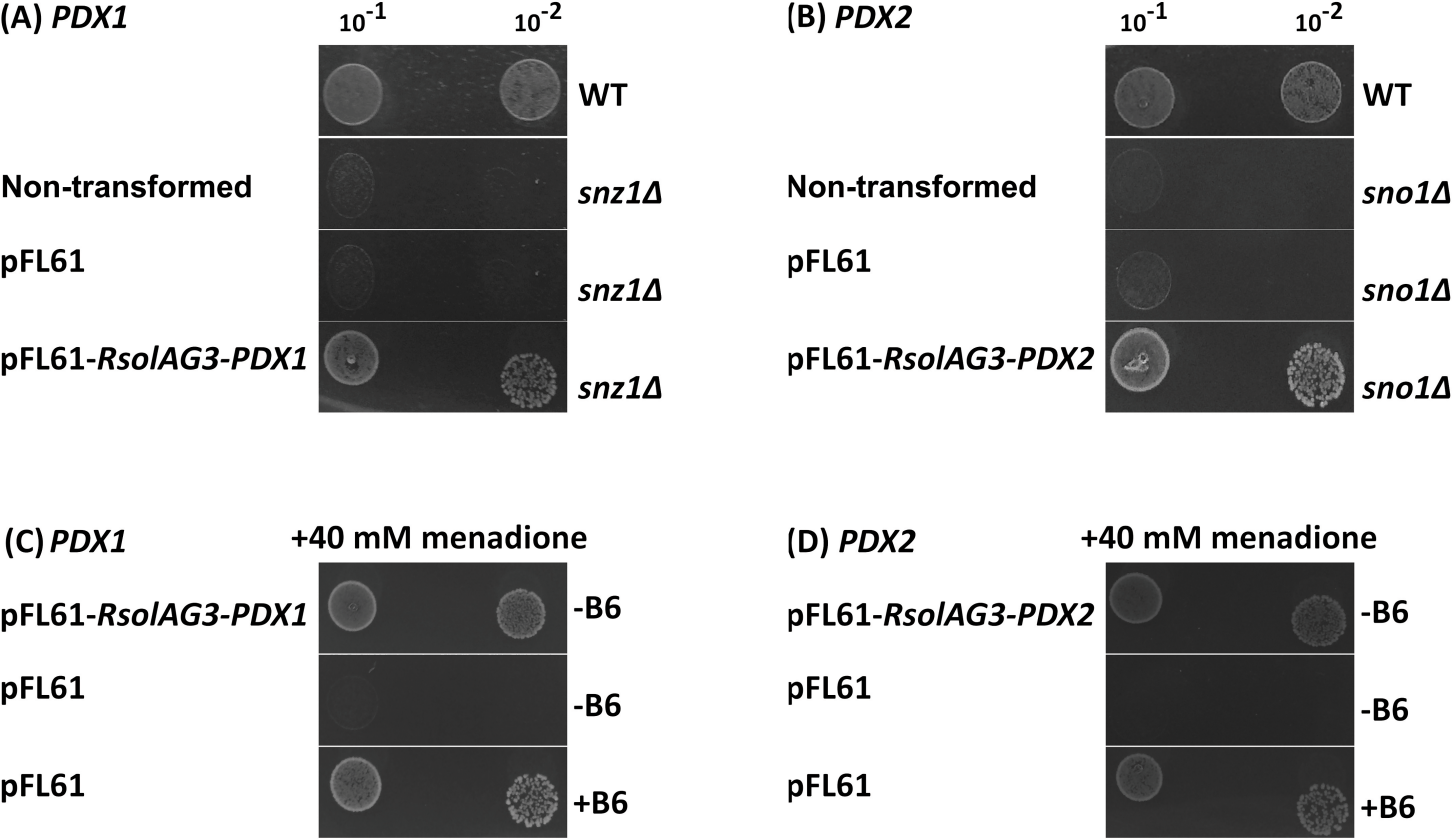
Complementation of the yeast *Δsnz1* and *Δsno1* mutants by *RsolPDX1* and *RsolPDX2*, respectively. Yeast cultures grown to exponential-phase followed by two serial 10-fold dilutions were placed onto vitamin B6-deficient SC (SC-B6) agar plates composed of (**A**) wild-type (WT), *Δsnz1*mutant, *Δsnz1* transformed with either pFL61 alone or the *RsolPDX1* expression vector (pFL61-*RsolPDX1*), (**B**) wild-type (WT), *Δsno1*mutant, *Δsno1* transformed with either pFL61 alone or the *RsolPDX2* expression vector (pFL61-*RsolPDX1*) (left colonies: 10^−1^ml^-1^ right colonies: 10^−2^ml^-1^). (**C**) *Δsnz1* mutant yeast transformed either with pFL61 alone or with the *RsolPDX1* expression vector (pFL61-*RsolPDX1*), and (**D**) *Δsno1* mutant yeast transformed either with pFL61 alone or with the *RsolPDX2* expression vector (pFL61-*RsolPDX2*) were both grown to the post-diauxic phase in SC-Uracil medium followed by a 180 min treatment with 40 mm menadione in the presence (+B6) or absence (-B6) of 2 μg ml^−1^ of vitamin B6. Following menadione treatment, two serial 10-fold dilutions using SC-Uracil medium (left colonies: 10^−1^ml^-1^, right colonies: 10^−2^ ml^-1^) were placed in SC-Uracil-B6 plates. Pictures were obtained after 3 d incubation at 30°C.

To determine whether *RsolPDX1* and *RsolPDX2* encode functional enzymes, the full coding sequence of each gene was cloned into the yeast expression vector pFL61 and transformed into *S*. *cerevisiae* strains defective in either *snz1* (yeast functional *PDX1* homolog; *Δsnz1*) or *sno1* (yeast functional *PDX2* homolog; *Δsno1*) and therefore unable to grow in media lacking vitamin B6 [31, 32]. Thus, growth of the transformed mutant *S*. *cerevisiae* cells on media lacking vitamin B6 will confirm the complementarity of the *R*. *solani* homologs to yeast vitamin B6 genes in addition to their role in vitamin B6 biosynthesis. On media not amended with pyridoxine, growth of *S*. *cerevisiae* strains Δsnz1 and Δsno1 expressing *RsolPDX1* and *RsolPDX2*, was evident (**Fig 2A and Fig 2B**). In contrast, the non-transformed strains or the empty vector-transformed Δsnz1 or Δsno1 mutant cells did not grow on media lacking pyridoxine. Complementation of the Δsnz1 mutant by *Rsol-PDX1* and Δsno1 mutant by *Rsol-PDX2* demonstrated that *Rsol-PDX1* and *Rsol-PDX2* are functional, and that they can replace the yeast SNZ1 and SNO1, respectively, implying that they are involved in *de novo* vitamin B6 biosynthesis.

Δsnz1 and Δsno1 display sensitivity to the superoxide generator menadione, and this sensitivity is corrected by the addition of vitamin B6 [31]. Thus, it is expected that vectors harbouring *Rsol-PDX1* or *Rsol-PDX2*, but not the mutants transformed with the empty vector, are resistant to menadione (**Fig 2C and Fig 2D).** Unsurprisingly, growth defect restoration of the empty vector-transformed mutants (*pFL61-Δsnz1*, *pFL61-Δsno1*) was achieved by the addition of 2 μg ml^−1^ of pyridoxine. In conclusion, these data indicate that that *Rsol-PDX1* and *Rsol-PDX2* confers resistance to ROS to the cells.

### Distinct expression of host and pathogen antioxidant genes in necrotic tissues and surrounding areas

**Fig 3:**
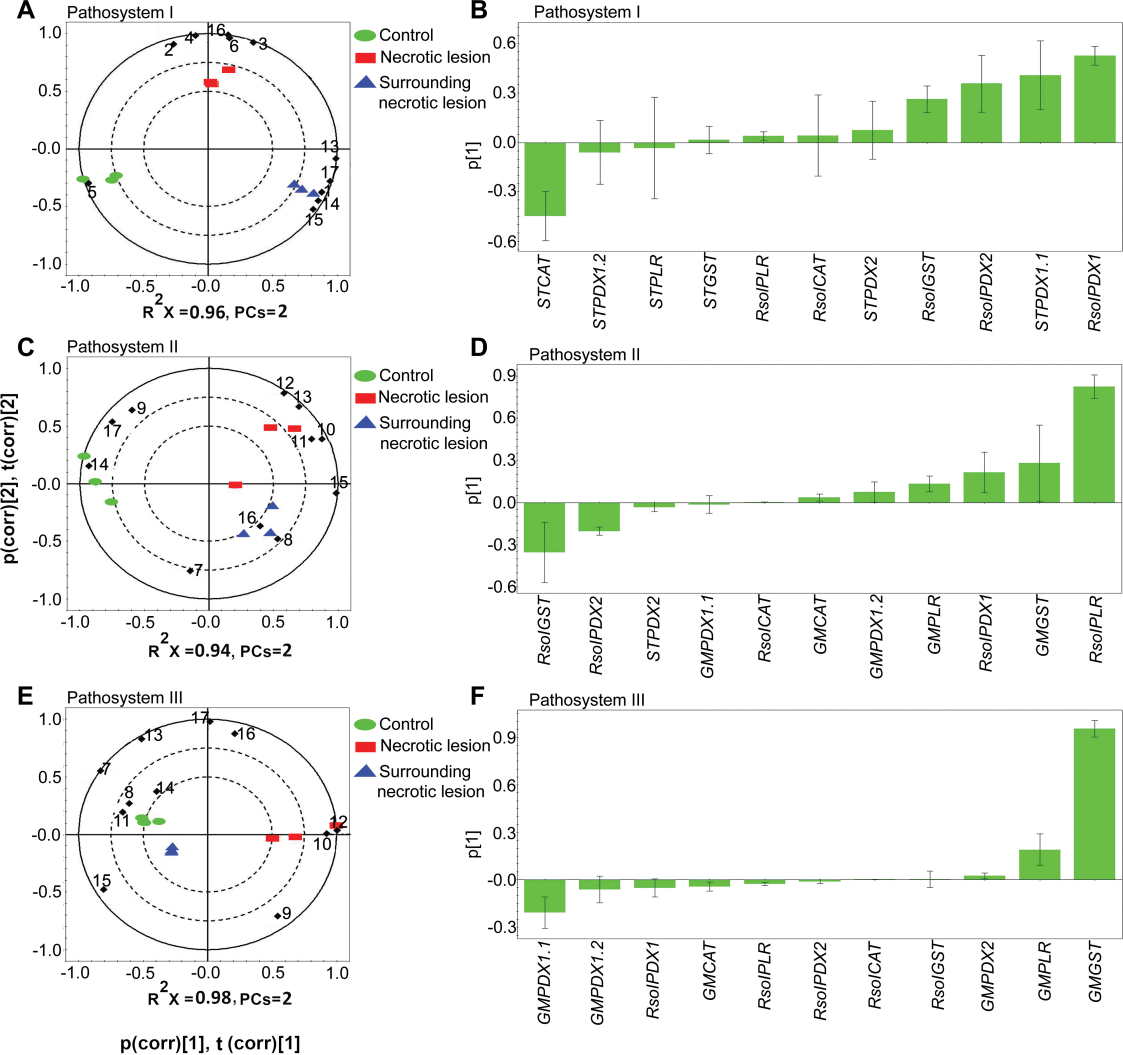
Principal component analysis (PCA) loading biplots for the effect of 11 antioxidant genes relative transcript abundance on control, infected and surrounding tissues in (**A**) pathosystem I, (**C**) pathosystem II, and (**E**) pathosystem III (P< 0.05). Principal component analysis (PCA) loading coefficient plots for the effect of 11 antioxidant genes relative transcript abundance on control, infected and surrounding tissues in (**B**) pathosystem I, (**D**) pathosystem II, and (**F**) pathosystem III. The scaled loading vectors pc (corr) and t (corr) are displayed for the first and the second component. Outer ellipses represent the Hotelling’s T^2^ at a 95% confidence interval. R^2^X represents the fraction of the sum of squares of the two principal components. *ST*: *Solanum tuberosum*, *GM*: *Glycine max*. Diamond represents genes. 1 = *STPDX1*.*1*, 2 = *STPDX1*.*2*, 3 = *STPDX2*, 4 = *STPLR*, 5 = *STCAT*, 6 = *STGST*, 7 = *GMPDX1*.*1*, 8 = *GMPDX1*.*2*, 9 = *GMPDX2*, 10 = *GMPLR*, 11 = *GMCAT*,12 = *GMGST*, 13 = *RsolPDX1*, 14 = *RsolPDX2*, 15 = *RsolPLR*, 16 = *RsolCAT*,17 = *RsolGST*.

For the discovery of trends of plant and fungal antioxidant encoding genes, in the three pathosystems, PCA revealed tight groups with no outliers (*P*<0.05) (**S1 Fig**). Antioxidant genes that influenced the separation of treatments (i.e., plant control, infected plant necrotic tissues and surrounding tissues) for each pathosystem were selected based on strong loading coefficients obtained from the loadings plots (**Fig 3**) and designated to have an effective antioxidant role in plant-pathogen interactions of each pathosystem. The strength of the correlations was pathosystem-dependent. Additionally, selected genes were highly associated with the distinct plant tissue type as revealed by the PCA- loading biplots (**Fig 3).**

### *R*. *solani* AG3 infection of potato sprouts significantly activates the fungal *RsolAG3-CAT* and *RsolAG3-GST* in addition to the *de novo* vitamin B6 genes of both the pathogen and the host

**Fig 4:**
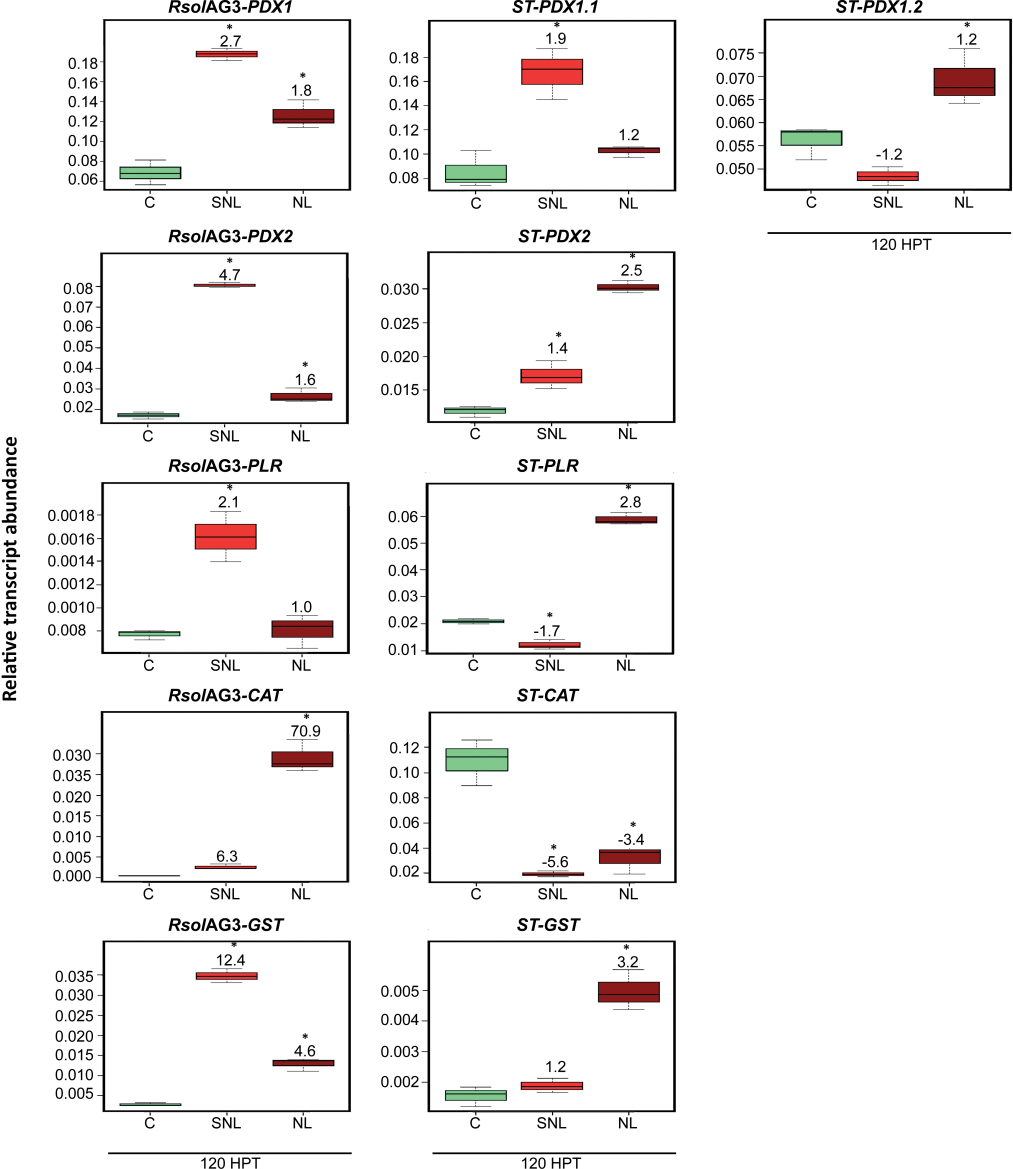
Relative transcript abundance of antioxidants in pathosystem I. Vitamin B6 (*PDX1*, *PDX2*, *and PLR*), *GST*, and *CAT* genes of the pathogen *R*. *solani* AG3 (*RsolAG3*) and the host, *Solanum tuberosum* (*ST*) in control and in infected and surrounding tissues at 120 HPI during *R*. *solani* AG3 and potato sprouts interaction. C: control; *R*. *solani* AG3 grown alone or potato sprouts inoculated with sterile with PDA SNL: tissue surrounding necrotic lesion. NL: necrotic lesion. Asterisks indicate significant relative transcript abundance ratios between the control and interaction using least significant difference (LSD) test (*P* < 0.05). Fold change is calculated in relation to the control at 120 HPI. *PDX1* and *PDX2*: Pyridoxine biosynthesis genes, *PLR*: *PYRIDOXAL REDUCTASE*, *CAT*: *CATALASE*, *GST*: *GLUTATHIONE S-TRANSFERASE*.

A remarkable gene upregulation of the fungal and plant vitamin B6 *de novo* pathway genes was observed during disease development of *R*. *solani* AG3 on potato sprouts. Based on PCA loading biplots and coefficients, the model showed that the loading coefficient values (p) of *RsolAG3-PDX1* (p, 54%) *ST-PDX1*.*1* (p, 40%), *RsolAG3-PDX2* (p, 35%), and *RsolAG3-GST* (p, 25%), were tightly linked with tissues of surrounding areas of necrotic lesions. On the other hand, the potato *CATALASE*, *ST-CAT* (p, -45%) was negatively associated with necrotic tissues and their surrounding areas (**Fig 3A and Fig 3B**). Significant fold increases of transcript abundances of the above fungal genes ranged from a minimum of 1.6 (*P =* 0.0029) to a maximum of 4.7 (*P*< 0.0001) depending on the gene and the plant’s area sampled (i.e., necrotic tissue vs surrounding or adjacent tissue) for *RsolAG3-PDX1* and *RsolAG3-PDX2* **(Fig 4**). Interestingly, the plant homologue *ST-PDX1*.*1* was differentially upregulated and tissue-dependent (**Fig 4**). *ST-PDX1*.*1* had a significant increase (1.9 folds; *P =* 0.0006) in only the tissues surrounding the necrotic area. Significant activation of fungal *RsolAG3-CAT* (70.9 folds; *P*< 0.0001) and *RsolAG3-GST* (12.4 folds; *P*< 0.0001) was observed in necrotic tissues and surrounding areas, respectively.

### Fungal-derived vitamin B6 genes and *RsolAG4-GST* are differentially regulated during soybean root and stem rot disease development caused by *R*. *solani* AG4

**Fig 5:**
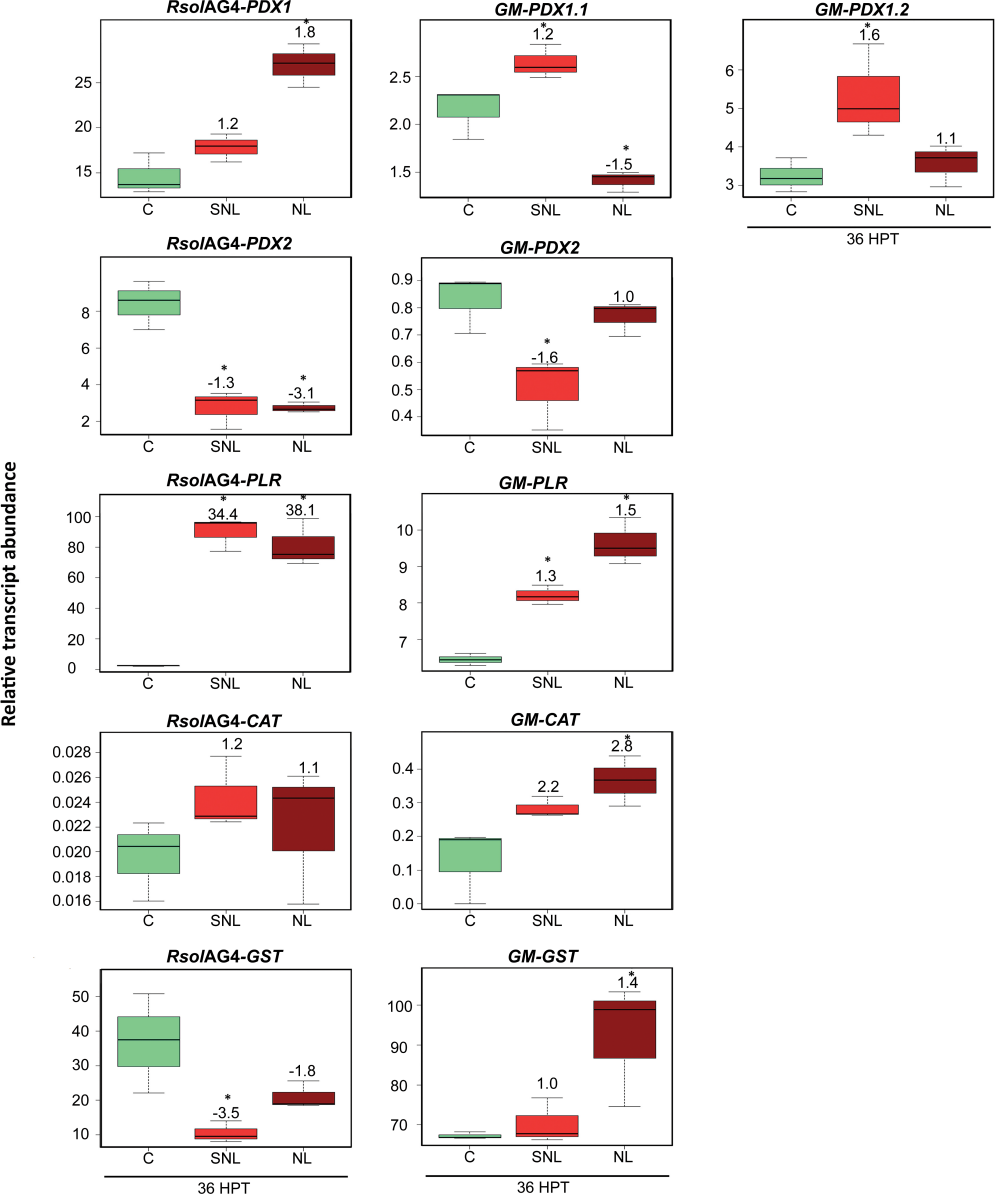
Relative transcript abundance of antioxidants in pathosystem II. Vitamin B6 (*PDX1*, *PDX2*, *and PLR*), *GST*, and *CAT* genes of both the pathogen *R*. *solani* AG4 (*RsolAG4*) and the host, *Glycine max*, (*GM*) in control and infected and surrounding tissues at 36 HPI during *R*. *solani* AG4 and soybean hypocotyls interaction. C: control; *R*. *solani* AG4 grown alone or soybean hypocotyl inoculated with sterile PDA. SNL: tissue surrounding necrotic lesion. NL: necrotic lesion. Asterisks indicate significant relative transcript abundance ratios between the control and the interaction using least significant difference (LSD) test (*P* < 0.05). Fold change is calculated in relation to the control at 36 HPI. *PDX1* and *PDX2*: Pyridoxine biosynthesis genes, *PLR*: *PYRIDOXAL REDUCTASE*, *CAT*: *CATALASE*, *GST*: *GLUTATHIONE S-TRANSFERASE*.

The fungal vitamin B6 genes along with *RsolAG4-GST* play a significant role in soybean*-R*. *solani* AG4 pathosystem compared to their plant’s counterpart during disease development (**Fig 3D**). A strong positive loading coefficient value (p, 80%) was observed for *RsolAG4-PLR* (**Fig 3D)** with significant upregulation of transcript abundance in necrotic tissues and surrounding areas by 38.1 fold (*P =* 0.0001) and 34.4 fold (*P =* 0.0001), respectively (**Fig 5**). *RsolAG4-PDX1* was moderately linked (p, 20%) with necrotic tissue showing an increase of transcript abundance by 1.8 fold (*P =* 0.0004) (**Fig 5**). However, *RsolAG4-GST* (p, -35%) and *RsolAG4-PDX2* (p, -20%) were negatively associated with necrotic tissue and surrounding areas. (**Fig 3C and Fig 3D**).

Among the plant-derived genes, *GM-GST (*p, 25%*)* and *GM-PLR* (p, 15%*)* were moderately linked with necrotic tissue (**Fig 3C and Fig 3D**). *GM-PLR* and *GM-GST* showed an increase of 1.5 (*P =* 0.0001) and 1.4 (*P =* 0.0183) folds, respectively, in necrotic tissue (**Fig 5**). The soybean *GM-CAT* encoding gene was positively upregulated in the necrotic tissues with 2.8 (*P =* 0.0109) fold increase (**Fig 5**). The *de novo* plant vitamin B6 genes showed an interesting pattern where both *GM-PDX2* and *GM-PDX1*.*1* were significantly downregulated by 1.6 (*P =* 0.0092) and 1.5 fold (*P =* 0.0036), respectively (**Fig 5**) while *GM-PDX1*.*2* was significantly upregulated in areas surrounding to necrotic tissues (1.6 fold; *P =* 0.0203) (**Fig 5**).

### Major plant-derived vitamin B6 genes and *GM-GST* play a prominent role during soybean RFB disease development caused by *R*. *solani* AG1-IA

**Fig 6:**
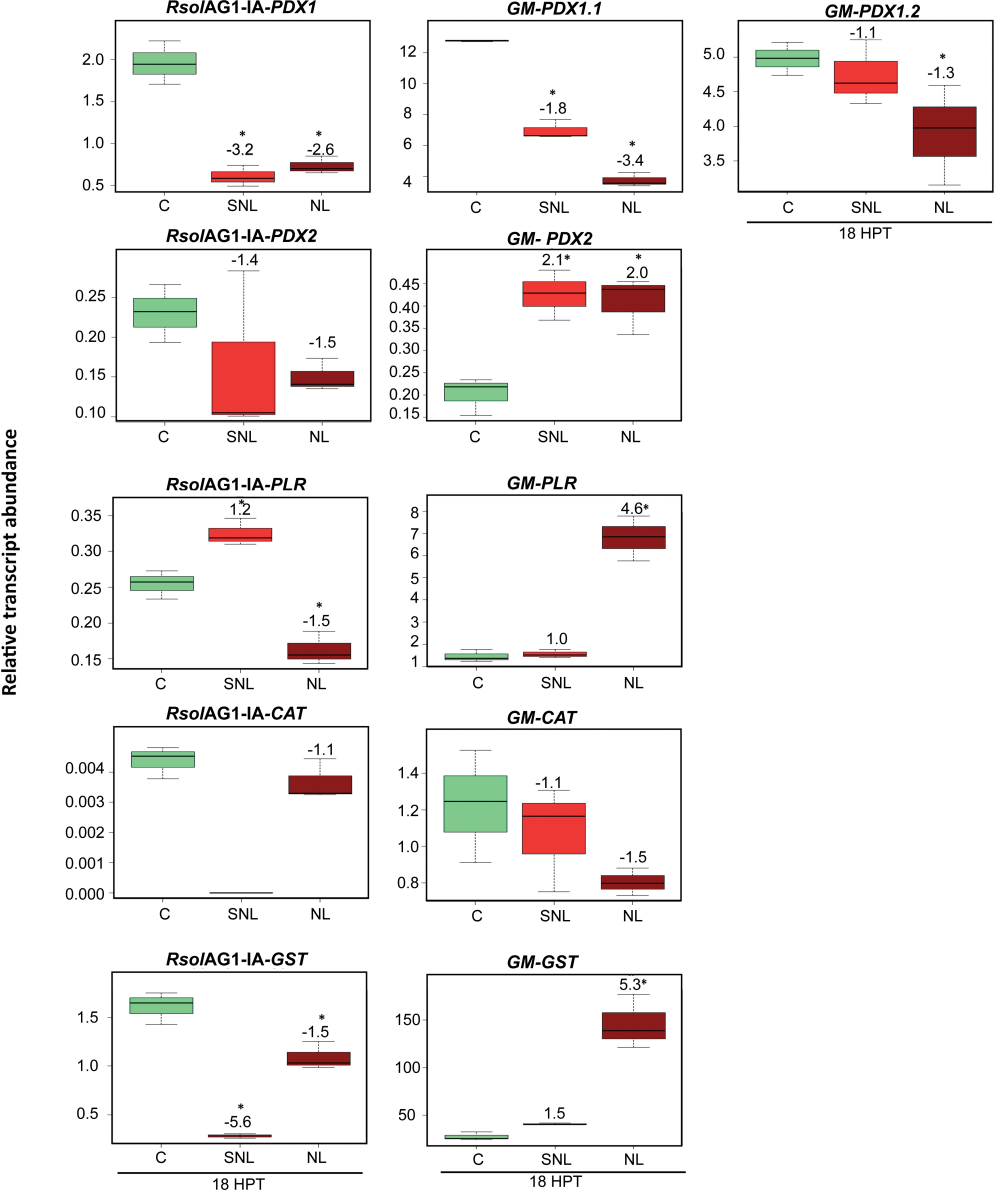
Relative transcript abundance of antioxidants in pathosystem III. Vitamin B6 (*PDX1*, *PDX2*, *and PLR*), *GST*, and *CAT* genes of both pathogen *R*. *solani* AG1-IA (*RsolAG1-IA*) and the host, *Glycine max* (*GM*) in control and in infected and surrounding tissues at 18 HPI during *R*. *solani* AG1-IA and soybean leaves interaction. C: control; *R*. *solani* AG1-IA grown alone or soybean leaf confronted with sterile PDA. SNL: tissue surrounding necrotic lesion. NL: necrotic lesion. Asterisk indicates significant relative transcript abundance ratios between the control and interaction using least significant difference (LSD) test (*P* < 0.05). Fold change is calculated in relation to the control at 18 HPI. *PDX1* and *PDX2*: Pyridoxine biosynthesis genes, *PLR*: *PYRIDOXAL REDUCTASE*, *CAT*: *CATALASE*, *GST*: *GLUTATHIONE S-TRANSFERASE*.

Upon fungal challenge, the relative transcripts abundance of the soybean *PYRIDOXAL REDUCTASE* (*GM-PLR)* and *GLUTATHIONE S-TRANSFERASE* (*GM-GST*) was highly upregulated whereas their corresponding fungal genes were notably downregulated (**Fig 6**). PCA analysis showed that leaf soybean *GM-GST* (p, 95%), and *GM-PLR* (p, 20%), were closely linked with the necrotic tissue, with significant increased abundance levels of *GM-GST* and *GM-PLR* by 5.3 (*P =* 0.0001) and 4.6 (*P<* 0.0001) fold, respectively (**Fig 6**). On the other hand, *GM-PDX1*.*1* (p, -20%) was negatively linked with necrotic tissue and surrounding areas (**Fig 3E and Fig 3F),** and displayed a significant downregulation with fold changes of 3.4 (*P<* 0.0001) and 1.8 (*P<* 0.0001) fold, respectively (**Fig 6**). Although not supported by high loading coefficient values in the PCA loading analysis, the fungal genes *RsolAG1-IA-PDX1* (one component of *de novo* vitamin B6 biosynthesis genes) and *Rso*AG1-IA-*GST* exhibited significant down-regulation with fold changes of 2.6 (*P =* 0.0001) and 1.5 (*P =* 0.0024), respectively, in necrotic tissues, and with fold changes of 3.2 (*P =* 0.0001) and 5.6 (*P<* 0.0001), respectively, in the surrounding areas (**Fig 6**).

### Accumulation of ROS in *R*. *solani* mycelia is related to transcriptional regulation of vitamin B6 machinery and other antioxidant genes

**Fig 7:**
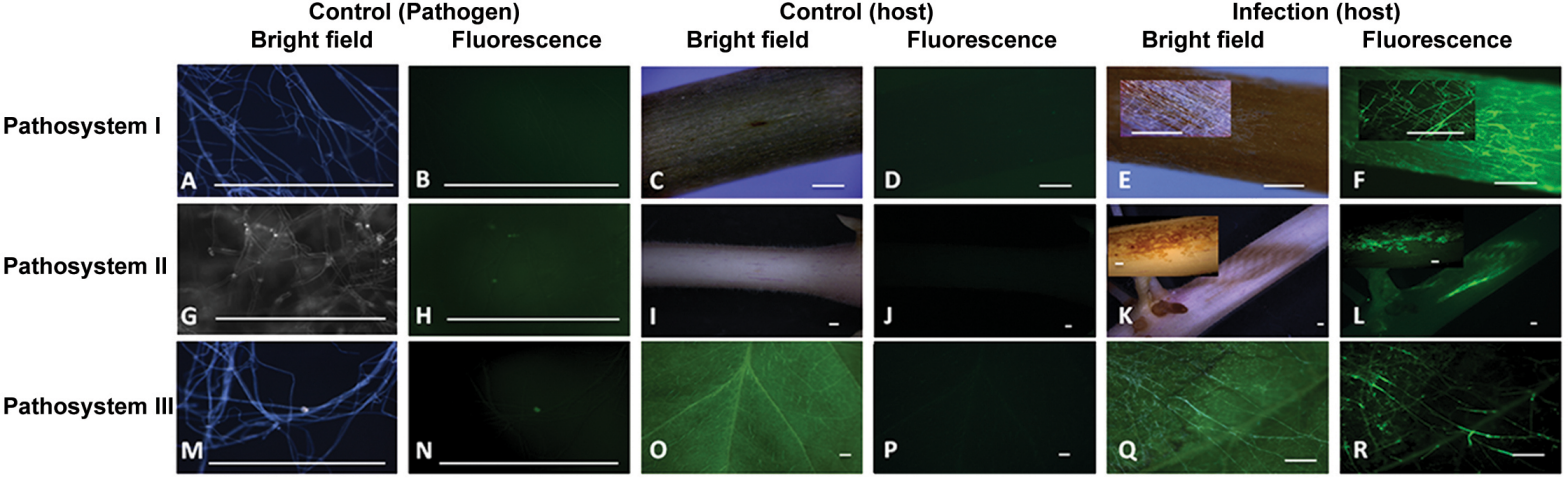
Intracellular production of ROS during disease development in pathosystems I-III. ROS production as visualized using H_2_DCF-DA in control and infected potato sprouts, soybean hypocotyls and leaves along with mycelia of control *R*. *solani* AG-3, AG-4, AG1-IA, grown on half-strength PDA overlaid with cellophane membranes. (**A-B**) Control mycelia with no evidence of endogenous ROS at 120 h, (**G-H**) 36 h, and (**M-N**) 18 h upon growth on PDA membrane overlaid with cellophane membranes. **(C-D)** Control *S*. *tuberosum* sprout, (**I-J**) *G*. *max* hypocotyl and (**O-P**) *G*. *max* leaf with no evidence of endogenous ROS. Mycelia of *R*. *solani* during disease development in pathosystems I-III (**Fig E-F, Fig K-L**, **and Fig Q-R**, respectively). Bar = 500 μm.

The presence of ROS in the fungal hyphae and plant tissues was detected by fluorescent H_2_DCF-DA and non-fluorescent DAB methods. Absence of fluorescence was observed in control hyphae grown on half-strength PDA (**Fig 7B, Fig 7H and Fig 7N**), while an intense green fluorescence was detected in *R*. *solani* AG3, AG4, and AG1-IA, hyphae during disease development (**Fig 7F, Fig 7L and Fig 7R**). Tissues of control and infected potato sprouts, soybean hypocotyls and leaves did not display any green fluorescence (**Fig 7D, Fig 7J and Fig 7P**) and (**Fig 7F, Fig 7L and Fig 7R**), respectively.

**Fig 8:**
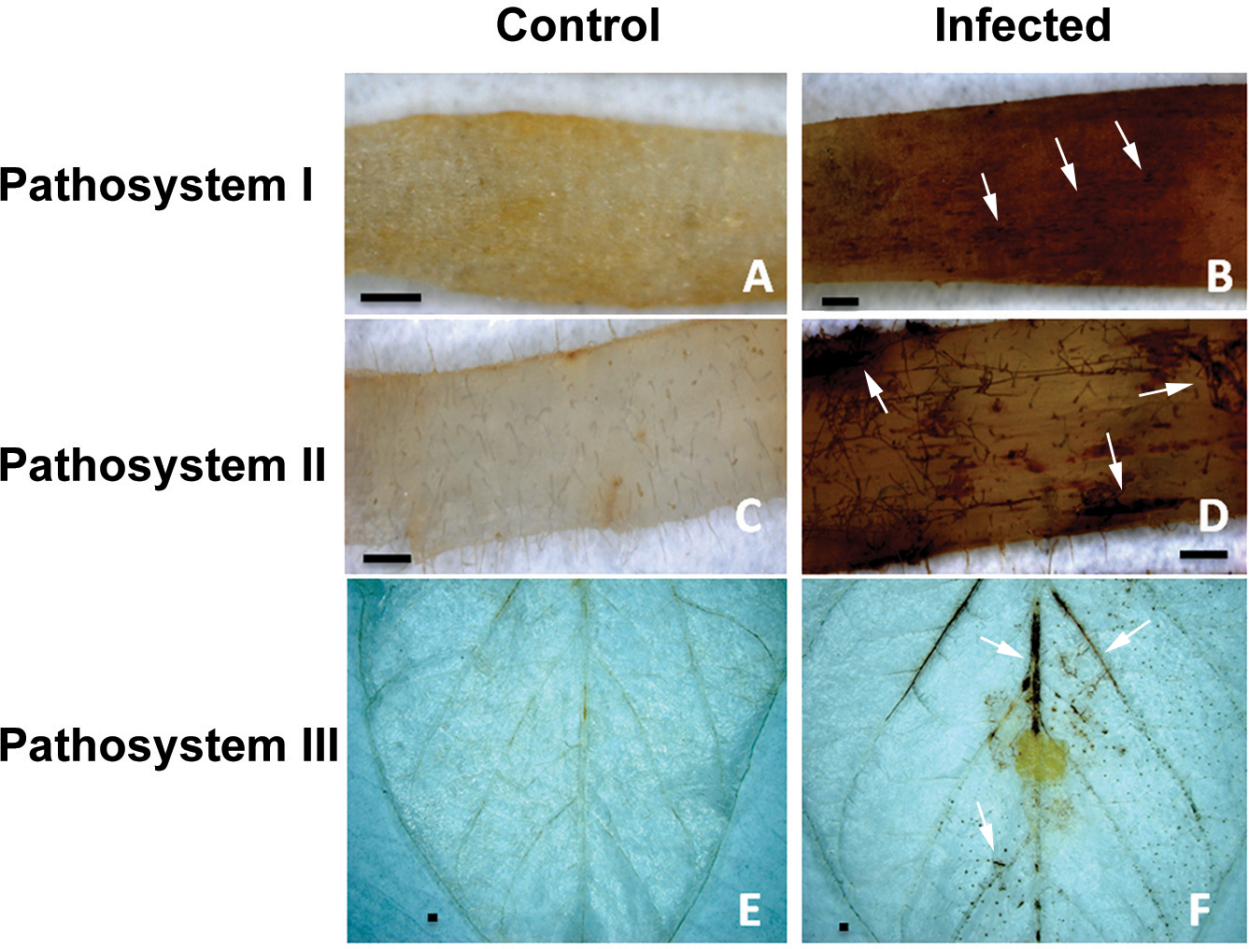
Microscopic detection of H_2_O_2_ accumulation using DAB staining during disease development in pathosystems I-III. H_2_O_2_ accumulation was visualized by DAB staining in control and infected potato sprouts and soybean hypocotyls and leaves. White arrows indicate dark brown precipitate developed at necrotic and surrounding areas. Control potato sprout (**A**), soybean hypocotyl (**C**), and soybean leaf (**E**) with no evidence of endogenous H_2_O_2_ at 120 HPT, 36h HPT, and 18h HPT, respectively. *R*. *solani* mycelia and *S*. *tuberosum* sprout (**B**), *G*. *max* hypocotyl (**D**), and *G*. *max* leaf (**F**) in pathosystems I-III with H_2_O_2_ accumulation. Bar = 500 μm.

To demonstrate whether the generation of ROS was promoted by *R*. *solani* infection, plant tissues were assayed *in situ* for the production of H_2_O_2_ in response to infection, using DAB staining. DAB oxidizes in the presence of H_2_O_2_ generating a dark brown precipitate in plant tissues [41]. Tissues of infected potato sprouts and soybean hypocotyls and leaves displayed a strong brown precipitate specifically at necrotic and surrounding areas (**Fig 8B, Fig 8D and Fig 8F**). However, control uninfected tissues did not show any browning (**Fig 8A, Fig 8C and Fig 8E**).

## Discussion

It is widely known that ROS management through the use of antioxidants is important for pathogenic microorganisms to prevent excessive oxidative stress [10, 11]. This research further supports our preceding study on the novel function of vitamin B6 genes in *R*. *solani* as an antioxidant stress protector against ROS that prevents expansion of oxidative stress [27]. Necrotrophic fungi can regulate intracellular levels of ROS for developmental and virulence purposes while the host uses ROS to hinder disease progression. A comprehensive understanding of plant responses to fungal pathogens exists [42, 43]. Gene expression studies focusing on oxidative stress responses from both the plant and the pathogen side during plant-*R*. *solani* interactions are very limited [28, 34]. This study is the first analysis of gene expression encoding vitamin B6 and several antioxidant genes in plant and fungal tissue of three pathosystems during interaction of different AG groups of *R*. *solani* with their respective hosts.

In a previous study, we fully characterized two vitamin B6 *de novo* biosynthetic pathway genes; *RsolPDX1* (**KF620111.1**) and *RsolPDX2* (**KF620112.1**) genes, and one gene, *RsolPLR* (**KJ395592.1**) of the vitamin B6 salvage biosynthetic pathway of *R*. *solani* AG3 [27]. The use of mutant yeasts and complementation with *PDX1* or *PDX2* proved to be a valuable tool in fungi [31, 32]. In this study, we confirmed that *R*. *solani* AG3 PDX1 and PDX2 are functionally exchangeable with yeast SNZ1 and SNO1, respectively, and are involved in the *de novo* vitamin B6 biosynthesis via successful complementation of the *S*. *cerevisiae* null mutant Δ*snz1* phenotype with *RsolAG3-PDX1*, and the null mutant *Δsno1* with *RsolAG3-PDX2*. Complementation of each of the *S*. *cerevisiae* null mutants (*Δsnz1* and *Δsno1*) with its respective *R*. *solani* AG3 gene restored their function implying their ability of *de novo* production of vitamin B6 in yeast. This also suggests that *RsolAG3-PDX1* and *RsolAG3-PDX2* can interact with their respective yeast counterpart to form the PDX1–PDX2 complex [44]. Similar to other fungi, our complementation assays validated the role *RsolAG3-PDX1* or *RsolAG3-PDX2* in oxidative stress resistance through the reversion of the menadione sensitivity of the *Δsnz1* or *Δsno1* mutant strains, respectively [31, 32]. Taken together, our results demonstrated that *R*. *solani* AG3 is probably autotrophic to vitamin B6. However, in order to provide direct evidence of autotrophy to vitamin B6, *in vitro* assays are required to demonstrate the capability of vitamin B6 synthesis by *R*. *solani* AG3 PDX1–PDX2 protein complex. Complementation assays for *PDX1*/*PDX2* of *R*. *solani* AG4 and AG1-IA were not attempted, as their respective genes are not characterized.

It is common knowledge that ROS is produced at the site of attempted invasion through an oxidative burst during disease development [9, 10, 28, 45]. Oxidative damage can be prevented in both the host and the pathogen when their antioxidant machinery is efficiently used [12, 46]. Both plants and fungi have ROS scavenging activities that detoxify ROS either directly through the action of enzymes such as; superoxide dismutase (*SOD*), catalase (*CAT*), glutathione peroxidase (*GPX*) and peroxiredoxins (*Prx*), or indirectly via vitamins like ascorbic acid (vitamin C), tocopherols (vitamin E), and vitamin B6 [23, 46–48].

To describe trends (positive or negative) between the plant’s and fungal antioxidants genes (total 11 antioxidant/pathosystem) in response to stress during plant-pathogen interactions of each pathosystem, it necessitates the use of multivariate analysis. The application of PCA revealed tight groups with no outliers. PCA loading biplots demonstrated that several trends could be observed between the relative transcript accumulation of antioxidant genes and the plant’s areas sampled (necrotic tissues or surrounding areas) in each pathosystem. These results indicate the distinctive differences in regulation of fungal-derived and plant-derived antioxidant genes of each pathosystem in response to *R*. *solani* attack of different AGs.

Our results clearly showed that ROS formation was induced in hyphal cells of *R*. *solani* AG1-IA, AG3 and AG4 during disease development on their respective hosts. ROS production was also reported in *R*. *solani* AG 2–2 IV and AG-8, serious pathogens of sugar beet and wheat, respectively [21, 28]. These findings support the notion that several AGs produce ROS. The induced ROS were also linked to the regulation of antioxidant genes and their collective possible role in oxidative stress alleviation. A strong brown precipitate was detected in tissues of infected potato sprouts and soybean hypocotyls and leaves. Accumulation of H_2_O_2_ in single cells followed by their death was reported in various plant-fungus interactions [40, 49–51]. These data confirm that *Rhizoctonia* disease development in potato sprouts and soybean hypocotyls and leaves provokes an oxidative stress in fungal hyphae and plant tissues specifically in necrotic areas and could be related to the oxidative status of the fungus as well as the plant.

The differential expression of antioxidant genes in the host and the pathogen is an important determinant of disease outcome and pathogenicity. ROS is under tight control during disease progression where sometimes it can lead to cell death of fungal and plant tissues. Alternatively, pathogen-induced ROS themselves can act as signalling molecule promoting development of fungal structures or resistance in plant cells [11, 28]. In our study, comparison of the differential gene expression across the three pathosystems was not attempted, because the method of inoculation and plant tissue types are different in each pathosystem, resulting in variable infection intensities and different gene expression. Instead, we focused on understanding the expression patterns of fungal and host antioxidant genes in each pathosystem. Our results showed that distinct expression patterns of fungal and host antioxidant genes were associated in necrotic tissues and their surrounding areas for each of the three *R*. *solani* pathosystems.

During *R*. *solani* AG3 infection of potato sprouts, significant gene expression of both fungal and plant vitamin B6 *de novo* pathway genes was observed. Genes of the vitamin B6 *de novo* pathway of *R*. *solani* and potato (homolog *ST-PDX1*.*1*) were highly induced in the surrounding tissue and to a lesser extent in the necrotic tissue. Interestingly, the potato *ST-PDX* genes (i.e., *ST-PDX1*.*1* and *ST-PDX 1*.*2* homologs of *ST-PDX1* and *ST-PDX 2)* are characterized according to their catalytic ability [52] and were expressed in all potato tissues [37]. The catalytic homolog *ST-PDX1*.*1*, but not the non-catalytic homolog *ST-PDX 1*.*2*, was induced in response to biotic and abiotic stress (this study, Moccand, Boycheva [52]). These results strongly indicate that *ST-PDX1*.*1* plays a role in disease development. It has been reported that *Arabidopsis* (AT-PDX1.2) acts as a pseudoenzyme that can have a role of a positive regulator of vitamin B6 abundance during abiotic stress [52]. Additionally, it was shown that potato *ST-PDX2* might be regulated by ST-PDX1.2, due to the unique (novel) ability of the latter to interact with *AT-PDX2* [37, 53]. In our study, the upregulation of *ST-PDX2* was not coupled by the upregulation of *ST-PDX1*.*2* during disease development. Whether ST-PDX1.2 acts as a positive regulator of vitamin B6 abundance during potato interaction with *R*. *solani* merits further studies.

Activation of some of the antioxidant genes was tissue specific. Higher expression of host genes (*ST-PDX2*, *ST-PLR* and *ST-GST*) compared to their counterpart in the pathogen in necrotic tissues was detected. Tissue specificity response is supported by the findings of Denslow, Walls [54]. The increase in the transcript abundance of *PDX1* was evident upon infection of tobacco leaves with *P*. *syringae pv*. *phaseolicola* at the region surrounding the infiltration area. However, levels of *PDX1* were lower in the infiltration area where hypersensitive response has developed [54].

In Pathosystem II, both fungal- and plant-derived antioxidant genes played a significant role during disease development where some of them were substantially induced while others were downregulated. Interestingly, fungal and host genes encoding *PLR*, a member of the vitamin B6 salvage biosynthetic pathways, were among the driving antioxidant force during soybean hypocotyl*-R*. *solani* interaction. Both fungal-derived and soybean-derived PLR encoding genes were expressed in the necrotic region as well as in the surrounding tissue, however *RsolAG4-PLR* was highly expressed. Similarly, in other studies, transcriptional regulation of PLR was substantially induced in response to accumulation of ROS in *R*. *solani* mycelia parasitized by a mycoparasite or exposed to different abiotic stressors [25, 27, 55]. The results of our study, clearly indicate the involvement of *RsolAG4-PLR* as an efficient quencher of ROS. On the other hand, the upregulation of soybean *PLR* (*GM-PLR*), but not at the same extent as that of the pathogen, in the same areas may imply that the plant may also be limiting ROS damage to the soybean tissue.

The interplay of ROS antioxidant genes produced by fungi and their hosts during their interaction was evident in *R*. *solani* AG1-IA-soybean leaves interaction (pathosystmem III). Major plant-derived antioxidant genes of soybean were upregulated in necrotic leaf tissues whereas the corresponding *R*. *solani* AG1-IA antioxidant genes showed notable downregulation. These results may possibly indicate that the elevation of antioxidant capacity of two of the three vitamin B6 genes, *GM-PDX2* and *GM-PLR* as well as the enzymatic antioxidant, *GLUTATHIONE S-TRANSFERASE* (*GM-GST)* in soybean leaves against *R*. *solani* AG1-IA increased their tolerance to the development of necrosis and suppressed the spread of the pathogen. These results are supported by the over expression of the antioxidant VB6 genes (i.e., *PDX)* in *Arabidopsis* that led to increased tolerance to oxidative stress [24, 56]. Equally *Arabidposis* mutants with defects in vitamin B6 *de novo* biosynthetic pathway (*PDX1*.*2* or *PDX1*.*3)* showed increased levels of disease of gray mold caused by *Botrytis cinerea* [57, 58]. Taken together, these results support the novel function of vitamin B6 genes as antioxidant stress protector against ROS to prevent oxidative stress.

In plant-necrotroph systems, the downregulation of antioxidant genes appears to play an important role during infection and initiation of the oxidative burst, leading to an overflow of ROS in infected areas. For example, in response to infection with *Botrytis cinerea*, leaves of ivy pelargonium (*Pelargonium peltatum*), showed a strong nitric oxide (NO) burst and H_2_O_2_ accumulation to arrest disease progression through NO-dependent reversible inhibition of catalase [59]. In our study, the plant’s *GmPDX1*.*1*, and *ST-CAT* in pathosystems I and III were substantially suppressed, respectively, which may lead to enhanced reactive oxygen species generation that typically accompanies infections caused by necrotrophs [60, 61].

## Conclusions

In conclusion, we confirmed the role of *R*. *solani* AG3 de novo vitamin B6 genes through yeast complementation assay. In our previous study [27], we provided evidence that vitamin B6 genes of the *de novo* and the salvage biosynthetic pathways function as antioxidants against oxidative stress induced by ROS chemical inducers [27]. In this study, we extend our research to examine whether genes of vitamin B6 machinery are also implicated as antioxidants in response to oxidative stress formed during plant-pathogen interactions. Our findings present new evidence on: (i) the co-expression of VB6 genes along with other well-known antioxidant genes (i.e., *CAT* and *GST*) in both the plant and pathogen side during their interaction, and (ii) the differential transcriptional regulation of the genes in infected necrotic tissues and their surrounding areas of three pathosystems involving different plants and different AGs of *R*. *solani*.

It is also likely that the requirement of Vitamin B6 genes of the salvage pathway differs among different plant species in response to pathogen infection. It appears that both the pathogen and host employ unique expression strategy depending on the type of interaction as well as the type of tissue affected. Hence, the study offers novel insights into the biological correlation and identification of ROS antioxidant genes that can be used in soybean and potato breeding programs for resistance against *R*. *solani*. Overall, we demonstrate that the co-expression and accumulation of certain plant and pathogen ROS-antioxidant related genes in each pathosystem are highlighted and might be critical during disease development from the plant’s point of view, and in pathogenicity and developing of infection structures from the fungal point of view. Future studies are underway to test whether different potato cultivars containing variable amounts of vitamin B6 [37] exhibit differential degrees of susceptibility to *R*. *solani* AG 3, and that this susceptibility is correlated with content and differential gene expression of vitamin B6.

## Supporting information

S1 Fig Principle component analysis score plots (PC1/PC2) for the effect of 11 antioxidant genes relative transcripts abundance on control, necrotic lesions and surrounding areas of necrotic lesions of Pathosystem I (**A**), II (**B**) and III (**C**). The ellipse represents the Hotelling T^2^ at a 95% confidence interval. Three biological replications were performed per treatment. Q2 (cum); cumulative fraction of the total variation of the X’s that can be predicted by the extracted components, R2X; the fraction of the sum of squares of the two principal components.
(TIF)

## References

[1] García VG, Onco MP, Susan VR. Biology and systematics of the form genus *Rhizoctonia*. Span J Agric Res. 2006;4(1):55–79.

[2] Buskila Y, Tsror Lahkim L, Sharon M, Teper-Bamnolker P, Holczer-Erlich O, Warshavsky S, et al Postharvest dark skin spots in potato tubers are an oversuberization response to *Rhizoctonia solani* infection. Phytopathology. 2011;101(4):436–44. doi: 10.1094/PHYTO-09-10-0251 . 21391824 10.1094/PHYTO-09-10-0251

[3] Fenille RC, De Souza NL, Kuramae EE. Characterization of *Rhizoctonia solani* associated with soybean in Brazil. Eur J Plant Pathol. 2002;108(8):783–92.

[4] Sneh B, Jabaji-Hare S, Neate S, Dijst G. Rhizoctonia species: taxonomy, molecular biology, ecology, pathology and disease control 1st ed: Springer Science & Business Media; 2013.

[5] Tsror L. Biology, Epidemiology and management of *Rhizoctonia solani* on potato. J Phytopathol. 2010;158(10):649–58. doi: 10.1111/j.1439-0434.2010.01671.x

[6] Ciampi MB, Meyer MC, Costa MJ, Zala M, McDonald BA, Ceresini PC. Genetic structure of populations of *Rhizoctonia solani* anastomosis group-1 IA from soybean in Brazil. Phytopathology. 2008;98(8):932–41. doi: 10.1094/PHYTO-98-8-0932 . 18943212 10.1094/PHYTO-98-8-0932

[7] Stetina KC, Stetina SR, Russin JS. Comparison of severity assessment methods for predicting yield loss to rhizoctonia foliar blight in soybean. Plant Dis. 2006;90(1):39–43. doi: 10.1094/pd-90-0039 10.1094/PD-90-0039 30786472

[8] Keijer J. The initial steps of the infection process in *Rhizoctonia solani* In: Sneh B JS, Neate S, Dijst G, editor. Rhizoctonia species: Taxonomy, molecular biology, ecology, pathology and disease control: Springer; 1996 p. 149–62.

[9] Torres MA. ROS in biotic interactions. Physiol Plant. 2010;138(4):414–29. doi: 10.1111/j.1399-3054.2009.01326.x 20002601 10.1111/j.1399-3054.2009.01326.x

[10] Alvarez ME, Pennell RI, Meijer PJ, Ishikawa A, Dixon RA, Lamb C. Reactive oxygen intermediates mediate a systemic signal network in the establishment of plant immunity. Cell. 1998;92(6):773–84. . 9529253 10.1016/s0092-8674(00)81405-1

[11] Barna B, Fodor J, Harrach BD, Pogany M, Kiraly Z. The Janus face of reactive oxygen species in resistance and susceptibility of plants to necrotrophic and biotrophic pathogens. Plant Physiol Biochem. 2012;59:37–43. doi: 10.1016/j.plaphy.2012.01.014 . 22321616 10.1016/j.plaphy.2012.01.014

[12] Apel K, Hirt H. Reactive oxygen species: metabolism, oxidative stress, and signal transduction. Annu Rev Plant Biol. 2004;55:373–99. doi: 10.1146/annurev.arplant.55.031903.141701 . 15377225 10.1146/annurev.arplant.55.031903.141701

[13] Asai S, Yoshioka H. Nitric oxide as a partner of reactive oxygen species participates in disease resistance to necrotrophic pathogen *Botrytis cinerea* in *Nicotiana benthamiana*. Mol Plant Microbe Interact. 2009;22(6):619–29. doi: 10.1094/MPMI-22-6-0619 19445587 10.1094/MPMI-22-6-0619

[14] Pietrowska E, Rozalska S, Kazmierczak A, Nawrocka J, Malolepsza U. Reactive oxygen and nitrogen (ROS and RNS) species generation and cell death in tomato suspension cultures-*Botrytis cinerea* interaction. Protoplasma. 2015;252(1):307–19. doi: 10.1007/s00709-014-0680-6 ; PubMed Central PMCID: PMCPMC4287684. 25064634 10.1007/s00709-014-0680-6 PMC4287684

[15] van Kan JA. Licensed to kill: the lifestyle of a necrotrophic plant pathogen. Trends Plant Sci. 2006;11(5):247–53. doi: 10.1016/j.tplants.2006.03.005 . 16616579 10.1016/j.tplants.2006.03.005

[16] Aliferis KA, Faubert D, Jabaji S. A metabolic profiling strategy for the dissection of plant defense against fungal pathogens. PLoS One. 2014;9(11):e111930 doi: 10.1371/journal.pone.0111930 ; PubMed Central PMCID: PMCPMC4219818. 25369450 10.1371/journal.pone.0111930 PMC4219818

[17] Aliferis KA, Jabaji S. FT-ICR/MS and GC-EI/MS metabolomics networking unravels global potato sprout's responses to *Rhizoctonia solani* infection. PLoS One. 2012;7(8):e42576 doi: 10.1371/journal.pone.0042576 ; PubMed Central PMCID: PMCPMC3411821. 22880040 10.1371/journal.pone.0042576 PMC3411821

[18] Foley RC, Gleason CA, Anderson JP, Hamann T, Singh KB. Genetic and genomic analysis of *Rhizoctonia solani* interactions with Arabidopsis; evidence of resistance mediated through NADPH oxidases. PLoS One. 2013;8(2):e56814 doi: 10.1371/journal.pone.0056814 ; PubMed Central PMCID: PMCPMC3581538. 23451091 10.1371/journal.pone.0056814 PMC3581538

[19] Keshavarz-Tohid V, Taheri P, Taghavi SM, Tarighi S. The role of nitric oxide in basal and induced resistance in relation with hydrogen peroxide and antioxidant enzymes. J Plant Physiol. 2016;199:29–38. doi: 10.1016/j.jplph.2016.05.005 . 27302004 10.1016/j.jplph.2016.05.005

[20] Nikraftar F, Taheri P, Falahati Rastegar M, Tarighi S. Tomato partial resistance to *Rhizoctonia solani* involves antioxidative defense mechanisms. Physiol Mol Plant P. 2013;81:74–83. doi: 10.1016/j.pmpp.2012.11.004

[21] Taheri P, Tarighi S. A survey on basal resistance and riboflavin-induced defense responses of sugar beet against *Rhizoctonia solani*. J Plant Physiol. 2011;168(10):1114–22. doi: 10.1016/j.jplph.2011.01.001 . 21269732 10.1016/j.jplph.2011.01.001

[22] Heller J, Tudzynski P. Reactive oxygen species in phytopathogenic fungi: signaling, development, and disease. Annu Rev Phytopathol. 2011;49:369–90. doi: 10.1146/annurev-phyto-072910-095355 . 21568704 10.1146/annurev-phyto-072910-095355

[23] Mooney S, Leuendorf JE, Hendrickson C, Hellmann H. Vitamin B6: a long known compound of surprising complexity. Molecules. 2009;14(1):329–51. doi: 10.3390/molecules14010329 . 19145213 10.3390/molecules14010329 PMC6253932

[24] Vanderschuren H, Boycheva S, Li KT, Szydlowski N, Gruissem W, Fitzpatrick TB. Strategies for vitamin B6 biofortification of plants: a dual role as a micronutrient and a stress protectant. Front Plant Sci. 2013;4:143 doi: 10.3389/fpls.2013.00143 ; PubMed Central PMCID: PMCPMC3659326. 23734155 10.3389/fpls.2013.00143 PMC3659326

[25] Chamoun R, Jabaji S. Expression of genes of *Rhizoctonia solani* and the biocontrol *Stachybotrys elegans* during mycoparasitism of hyphae and sclerotia. Mycologia. 2011;103(3):483–93. doi: 10.3852/10-235 . 21193602 10.3852/10-235

[26] Gkarmiri K, Finlay RD, Alstrom S, Thomas E, Cubeta MA, Hogberg N. Transcriptomic changes in the plant pathogenic fungus *Rhizoctonia solani* AG-3 in response to the antagonistic bacteria *Serratia proteamaculans* and *Serratia plymuthica*. BMC Genomics. 2015;16:630 doi: 10.1186/s12864-015-1758-z ; PubMed Central PMCID: PMCPMC4546130. 26296338 10.1186/s12864-015-1758-z PMC4546130

[27] Samsatly J, Chamoun R, Gluck-Thaler E, Jabaji S. Genes of the de novo and salvage biosynthesis pathways of vitamin b6 are regulated under oxidative stress in the plant pathogen *Rhizoctonia solani*. Front Microbiol. 2015;6:1429 doi: 10.3389/fmicb.2015.01429 ; PubMed Central PMCID: PMCPMC4700284. 26779127 10.3389/fmicb.2015.01429 PMC4700284

[28] Foley RC, Kidd BN, Hane JK, Anderson JP, Singh KB. Reactive oxygen species play a role in the infection of the necrotrophic fungi, *Rhizoctonia solani* in wheat. PLoS One. 2016;11(3):e0152548 doi: 10.1371/journal.pone.0152548 ; PubMed Central PMCID: PMCPMC4816451. 27031952 10.1371/journal.pone.0152548 PMC4816451

[29] Minet M, Dufour ME, Lacroute F. Complementation of *Saccharomyces cerevisiae* auxotrophic mutants by *Arabidopsis thaliana* cDNAs. The Plant Journal. 1992;2(3):417–22. 1303803 10.1111/j.1365-313x.1992.00417.x

[30] Schiestl RH, Gietz RD. High efficiency transformation of intact yeast cells using single stranded nucleic acids as a carrier. Curr Genet. 1989;16(5–6):339–46. doi: 10.1007/bf00340712 2692852 10.1007/BF00340712

[31] Rodriguez-Navarro S, Llorente B, Rodriguez-Manzaneque MT, Ramne A, Uber G, Marchesan D, et al Functional analysis of yeast gene families involved in metabolism of vitamins B1 and B6. Yeast. 2002;19(14):1261–76. doi: 10.1002/yea.916 . 12271461 10.1002/yea.916

[32] Benabdellah K, Azcon-Aguilar C, Valderas A, Speziga D, Fitzpatrick TB, Ferrol N. GintPDX1 encodes a protein involved in vitamin B6 biosynthesis that is up-regulated by oxidative stress in the arbuscular mycorrhizal fungus *Glomus intraradices*. New Phytol. 2009;184(3):682–93. doi: 10.1111/j.1469-8137.2009.02978.x . 19674326 10.1111/j.1469-8137.2009.02978.x

[33] Chamoun R, Samsatly J, Pakala SB, Cubeta MA, Jabaji S. Suppression subtractive hybridization and comparative expression of a pore-forming toxin and glycosyl hydrolase genes in Rhizoctonia solani during potato sprout infection. Molecular Genetics and Genomics. 2015;290(3):877–900. doi: 10.1007/s00438-014-0962-x 25472038 10.1007/s00438-014-0962-x

[34] Copley TR, Aliferis KA, Kliebenstein DJ, Jabaji SH. An integrated RNAseq-1H NMR metabolomics approach to understand soybean primary metabolism regulation in response to *Rhizoctonia* foliar blight disease. BMC Plant Biol. 2017;17(1):84 doi: 10.1186/s12870-017-1020-8 ; PubMed Central PMCID: PMCPMC5408482. 28449662 10.1186/s12870-017-1020-8 PMC5408482

[35] Chamoun R, Aliferis KA, Jabaji SH. Characterization and transcriptional regulation of *Stachybotrys elegans* mitogen-activated-protein kinase gene smkA following mycoparasitism and starvation conditions. Curr Genet. 2013;59(1–2):43–54. doi: 10.1007/s00294-012-0386-2 . 23271388 10.1007/s00294-012-0386-2

[36] Zhao S, Fernald RD. Comprehensive algorithm for quantitative real-time polymerase chain reaction. J Comp Biol. 2005;12(8):1047–64. 10.1089/cmb.2005.12.1047 PMC2716216 16241897

[37] Mooney S, Chen L, Kuhn C, Navarre R, Knowles NR, Hellmann H. Genotype-specific changes in vitamin B6 content and the PDX family in potato. Biomed Res Int. 2013;2013:389723 doi: 10.1155/2013/389723 ; PubMed Central PMCID: PMCPMC3732595. 23971030 10.1155/2013/389723 PMC3732595

[38] Libault M, Thibivilliers S, Bilgin D, Radwan O, Benitez M, Clough S, et al Identification of four soybean reference genes for gene expression normalization. The Plant Genome. 2008;1(1):44–54.

[39] Johanson A, Turner HC, McKay GJ, Brown AE. A PCR-based method to distinguish fungi of the rice sheath-blight complex, *Rhizoctonia solani*, *R*. *oryzae* and *R*. *oryzae*-sativae. FEMS Microbiol Lett. 1998;162(2):289–94. 9627963 10.1111/j.1574-6968.1998.tb13011.x

[40] Pogany M, von Rad U, Grun S, Dongo A, Pintye A, Simoneau P, et al Dual roles of reactive oxygen species and NADPH oxidase RBOHD in an *Arabidopsis*-*Alternaria* pathosystem. Plant Physiol. 2009;151(3):1459–75. doi: 10.1104/pp.109.141994 ; PubMed Central PMCID: PMCPMC2773049. 19726575 10.1104/pp.109.141994 PMC2773049

[41] Thordal‐Christensen H, Zhang Z, Wei Y, Collinge DB. Subcellular localization of H_2_O_2_ in plants. H_2_O_2_ accumulation in papillae and hypersensitive response during the barley—powdery mildew interaction. Plant J. 1997;11(6):1187–94.

[42] Conrath U. Priming of induced plant defense responses. Adv Bot Res. 2009;51:361–95. doi: 10.1016/s0065-2296(09)51009-9

[43] Glazebrook J. Contrasting mechanisms of defense against biotrophic and necrotrophic pathogens. Annu Rev Phytopathol. 2005;43:205–27. doi: 10.1146/annurev.phyto.43.040204.135923 . 16078883 10.1146/annurev.phyto.43.040204.135923

[44] Gengenbacher M, Fitzpatrick TB, Raschle T, Flicker K, Sinning I, Muller S, et al Vitamin B6 biosynthesis by the malaria parasite *Plasmodium falciparum*: biochemical and structural insights. J Biol Chem. 2006;281(6):3633–41. doi: 10.1074/jbc.M508696200 . 16339145 10.1074/jbc.M508696200

[45] Lehtonen MJ, Somervuo P, Valkonen JP. Infection with *Rhizoctonia solani* induces defense genes and systemic resistance in potato sprouts grown without light. Phytopathology. 2008;98(11):1190–8. doi: 10.1094/PHYTO-98-11-1190 . 18943407 10.1094/PHYTO-98-11-1190

[46] Sharma P, Jha AB, Dubey RS, Pessarakli M. Reactive oxygen species, oxidative damage, and antioxidative defense mechanism in plants under stressful conditions. J Bot 2012;2012:1–26. doi: 10.1155/2012/217037

[47] Breitenbach M, Weber M, Rinnerthaler M, Karl T, Breitenbach-Koller L. Oxidative stress in fungi: its function in signal transduction, interaction with plant hosts, and lignocellulose degradation. Biomolecules. 2015;5(2):318–42. doi: 10.3390/biom5020318 25854186 10.3390/biom5020318 PMC4496675

[48] Mylona PV, Polidoros AN. ROS regulation of antioxidant genes. Reactive Oxygen Species and Antioxidants in Higher Plants. 2010:101.

[49] Asselbergh B, Curvers K, Franca SC, Audenaert K, Vuylsteke M, Van Breusegem F, et al Resistance to *Botrytis cinerea* in sitiens, an abscisic acid-deficient tomato mutant, involves timely production of hydrogen peroxide and cell wall modifications in the epidermis. Plant Physiol. 2007;144(4):1863–77. doi: 10.1104/pp.107.099226 ; PubMed Central PMCID: PMCPMC1949893. 17573540 10.1104/pp.107.099226 PMC1949893

[50] Chung KR. Stress response and pathogenicity of the necrotrophic fungal pathogen *Alternaria alternata*. Scientifica (Cairo). 2012;2012:635431 doi: 10.6064/2012/635431 ; PubMed Central PMCID: PMCPMC3820455. 24278721 10.6064/2012/635431 PMC3820455

[51] Laluk K, Mengiste T. Necrotroph attacks on plants: wanton destruction or covert extortion? Arabidopsis Book. 2010;8:e0136 doi: 10.1199/tab.0136 ; PubMed Central PMCID: PMCPMC3244965. 22303261 10.1199/tab.0136 PMC3244965

[52] Moccand C, Boycheva S, Surriabre P, Tambasco-Studart M, Raschke M, Kaufmann M, et al The pseudoenzyme PDX1.2 boosts vitamin B6 biosynthesis under heat and oxidative stress in *Arabidopsis*. J Biol Chem. 2014;289(12):8203–16. doi: 10.1074/jbc.M113.540526 ; PubMed Central PMCID: PMCPMC3961649. 24505140 10.1074/jbc.M113.540526 PMC3961649

[53] Wagner S, Bernhardt A, Leuendorf JE, Drewke C, Lytovchenko A, Mujahed N, et al Analysis of the *Arabidopsis* rsr4-1/pdx1-3 mutant reveals the critical function of the PDX1 protein family in metabolism, development, and vitamin B6 biosynthesis. Plant Cell. 2006;18(7):1722–35. doi: 10.1105/tpc.105.036269 ; PubMed Central PMCID: PMCPMC1488916. 16766694 10.1105/tpc.105.036269 PMC1488916

[54] Denslow SA, Walls AA, Daub ME. Regulation of biosynthetic genes and antioxidant properties of vitamin B6 vitamers during plant defense responses. Physiol Mol Plant P. 2005;66(6):244–55. doi: 10.1016/j.pmpp.2005.09.004

[55] Morissette DC, Dauch A, Beech R, Masson L, Brousseau R, Jabaji-Hare S. Isolation of mycoparasitic-related transcripts by SSH during interaction of the mycoparasite *Stachybotrys elegans* with its host *Rhizoctonia solani*. Curr Genet. 2008;53(2):67–80. doi: 10.1007/s00294-007-0166-6 . 18058103 10.1007/s00294-007-0166-6

[56] Raschke M, Boycheva S, Crevecoeur M, Nunes-Nesi A, Witt S, Fernie AR, et al Enhanced levels of vitamin B(6) increase aerial organ size and positively affect stress tolerance in *Arabidopsis*. Plant J. 2011;66(3):414–32. doi: 10.1111/j.1365-313X.2011.04499.x . 21241390 10.1111/j.1365-313X.2011.04499.x

[57] Zhang Y, Jin X, Ouyang Z, Li X, Liu B, Huang L, et al Vitamin B6 contributes to disease resistance against *Pseudomonas syringae* pv. tomato DC3000 and *Botrytis cinerea* in *Arabidopsis thaliana*. J Plant Physiol. 2015;175:21–5. doi: 10.1016/j.jplph.2014.06.023 . 25460872 10.1016/j.jplph.2014.06.023

[58] Zhang Y, Liu B, Li X, Ouyang Z, Huang L, Hong Y, et al The de novo biosynthesis of vitamin B6 is required for disease resistance against *Botrytis cinerea* in tomato. Mol Plant Microbe Interact. 2014;27(7):688–99. doi: 10.1094/MPMI-01-14-0020-R . 24678833 10.1094/MPMI-01-14-0020-R

[59] Floryszak-Wieczorek J, Arasimowicz M, Milczarek G, Jelen H, Jackowiak H. Only an early nitric oxide burst and the following wave of secondary nitric oxide generation enhanced effective defence responses of pelargonium to a necrotrophic pathogen. New Phytol. 2007;175(4):718–30. doi: 10.1111/j.1469-8137.2007.02142.x . 17688587 10.1111/j.1469-8137.2007.02142.x

[60] Able AJ. Role of reactive oxygen species in the response of barley to necrotrophic pathogens. Protoplasma. 2003;221(1):137–43. 12768351 10.1007/s00709-002-0064-1

[61] Unger C, Kleta S, Jandl G, Tiedemann Av. Suppression of the Defence‐Related Oxidative Burst in Bean Leaf Tissue and Bean Suspension Cells by the Necrotrophic Pathogen Botrytis cinerea. Journal of Phytopathology. 2005;153(1):15–26.

