# Supplementary figures and images for: Antioxidant genes of plants and fungal pathogens are distinctly regulated during disease development in different Rhizoctonia solani pathosystems
Source: PLoS One. 2018 Feb 21;13(2):e0192682. doi: 10.1371/journal.pone.0192682 (PMC5821333; doi:10.1371/journal.pone.0192682)

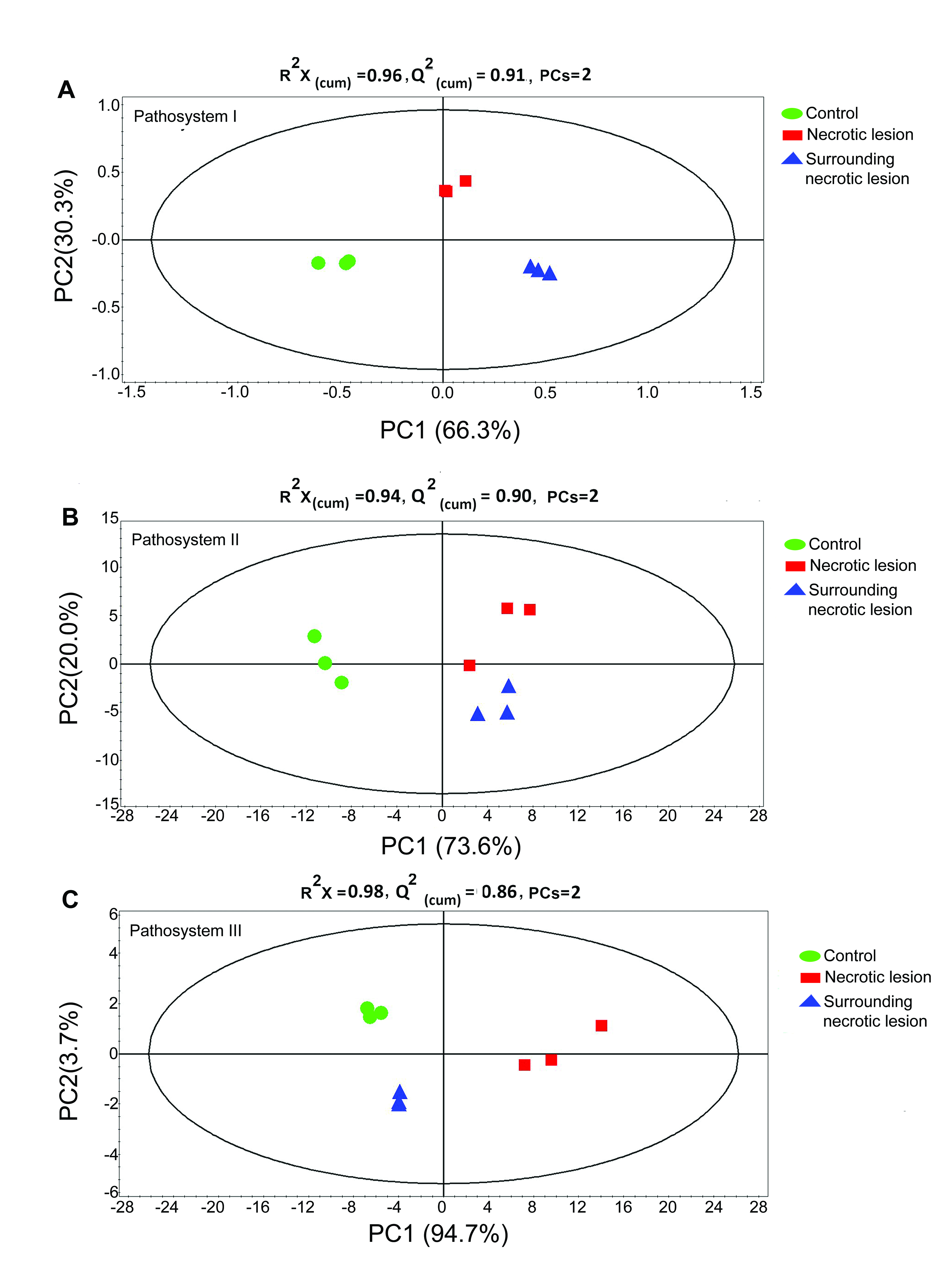

Supplement: S1 Fig — Principle component analysis score plots (PC1/PC2) for the effect of 11 antioxidant genes relative transcripts abundance on control, necrotic lesions and surrounding areas of necrotic lesions of Pathosystem I (A), II (B) and III (C). The ellipse represents the Hotelling T2 at a 95% confidence interval. Three biological replications were performed per treatment. Q2 (cum); cumulative fraction of the total variation of the X’s that can be predicted by the extracted components, R2X; the fraction of the sum of squares of the two principal components. (TIF) [file pone.0192682.s001.tif]
